# Identification of MYH9 as a key regulator for synoviocyte migration and invasion through secretome profiling

**DOI:** 10.1136/ard-2022-223625

**Published:** 2023-05-15

**Authors:** Saseong Lee, Eunbyeol Choi, Sehyun Chae, Jung Hee Koh, Yoolim Choi, Jung Gon Kim, Seung-Ah Yoo, Daehee Hwang, Wan-Uk Kim

**Affiliations:** 1 Center for Integrative Rheumatoid Transcriptomics and Dynamics, The Catholic University of Korea, Seoul, The Republic of Korea; 2 Department of Biomedicine & Health Sciences, College of Medicine, The Catholic University of Korea, Seoul, The Republic of Korea; 3 Neurovascular Unit Research Group, Korea Brain Research Institute, Daegu, The Republic of Korea; 4 Department of Internal Medicine, The Catholic University of Korea, School of Medicine, Seoul, The Republic of Korea; 5 Department of Biological Sciences, Seoul National University, Seoul, The Republic of Korea; 6 Department of Internal Medicine, Inje University Ilsan Paik Hospital, Goyang, The Republic of Korea; 7 Department of Medical Life Sciences, The Catholic University of Korea, Seoul, The Republic of Korea

**Keywords:** arthritis, rheumatoid, autoimmune diseases, synovitis, synovial fluid

## Abstract

**Objectives:**

‘Invasive pannus’ is a pathological hallmark of rheumatoid arthritis (RA). This study aimed to investigate secretome profile of synovial fibroblasts of patients with RA (RA-FLSs), a major cell type comprising the invasive pannus.

**Methods:**

Secreted proteins from RA-FLSs were first identified using liquid chromatography-tandem mass spectrometry analysis. Ultrasonography was performed for affected joints to define synovitis severity at the time of arthrocentesis. Expression levels of myosin heavy chain 9 (MYH9) in RA-FLSs and synovial tissues were determined by ELISA, western blot analysis and immunostaining. A humanised synovitis model was induced in immuno-deficient mice.

**Results:**

We first identified 843 proteins secreted from RA-FLSs; 48.5% of the secretome was associated with pannus-driven pathologies. Parallel reaction monitoring analysis of the secretome facilitated discovery of 16 key proteins related to ‘invasive pannus’, including MYH9, in the synovial fluids, which represented synovial pathology based on ultrasonography and inflammatory activity in the joints. Particularly, MYH9, a key protein in actin-based cell motility, showed a strong correlation with fibroblastic activity in the transcriptome profile of RA synovia. Moreover, MYH9 expression was elevated in cultured RA-FLSs and RA synovium, and its secretion was induced by interleukin-1β, tumour necrosis factor α, toll-like receptor ligation and endoplasmic reticulum stimuli. Functional experiments demonstrated that MYH9 promoted migration and invasion of RA-FLSs in vitro and in a humanised synovitis model, which was substantially inhibited by blebbistatin, a specific MYH9 inhibitor.

**Conclusions:**

This study provides a comprehensive resource of the RA-FLS-derived secretome and suggests that MYH9 represents a promising target for retarding abnormal migration and invasion of RA-FLSs.

**WHAT IS ALREADY KNOWN ON THIS TOPIC**

- ‘Invasive pannus’ is a pathological hallmark of rheumatoid arthritis (RA).
- Various proteins secreted from fibroblast-like synoviocytes of patients with RA (RA-FLSs) critically drive such pathology; however, they have not been globally and systematically investigated.

**WHAT THIS STUDY ADDS**

- We newly identified the 16 key proteins from RA-FLSs, which well represented pannus-driven pathologies, via global secretome analysis and named the proteins ‘synoviocyte secretome signature’ (SSS).
- Among the 16 proteins of SSS, we particularly focused on myosin heavy chain 9 (MYH9), a component of actin-based cell motility, as a key regulator of ‘invasive pannus’ based on its novelty and the excellent representativeness of pannus pathology.
- Interestingly, MYH9 showed a strong correlation with fibroblastic activity in the transcriptome profile of RA synovia.
- Moreover, MYH9 was highly expressed in cultured RA-FLSs and RA synovium, and it promoted migration and invasion of RA-FLSs in vitro and in a severe combined immunodeficiency mouse-xenograft (RA-FLSs) model.
- Of note, blebbistatin, an inhibitor of MYH9, substantially mitigated the aggressiveness of RA-FLSs in vitro and in vivo, thereby leading to less cartilage destruction in a humanised synovitis model and in mouse models of chronic inflammatory arthritis.

**HOW THIS STUDY MIGHT AFFECT RESEARCH, PRACTICE OR POLICY**

- We provide a comprehensive resource of RA-FLS-derived secretome, which can be used as a fundamental resource in diverse studies and can ultimately help to discover novel regulators for pathological processes mediated by secreted proteins from RA-FLSs.
- Our study also demonstrates that MYH9 is essential to migration and invasion of RA-FLSs and that MYH9 inhibition by blebbistatin could be an effective strategy to retard FLS-driven migration/invasion and cartilage degradation in RA.

## Introduction

Rheumatoid arthritis (RA) is characterised by synovial proliferation and extensive angiogenesis called pannus formation,^1^ which has been considered a pathological hallmark of RA. In particular, fibroblast-like synoviocytes (FLSs) exhibiting resistance to apoptosis, abnormal proliferation and pro-migratory capacity are a major cell type comprising the invasive pannus.^2^ The FLSs from patients with RA (RA-FLSs) secrete various disease-aggravating factors. These factors are crucial to the pathogenesis of RA owing to their roles in perpetuation of synovial inflammation, chemoattraction of immune cells, promotion of neo-vascularisation, generation of auto-antigens and infliction of permanent damage to joints.^3^ Therefore, a variety of secreted molecules from RA-FLSs may serve as useful biomarkers that represent disease severity of RA and can potentially represent therapeutic targets.^4^ Nevertheless, global and systematic profiles of secretory molecules originating from RA-FLSs remain to be clarified.

Identification of new molecules derived from the ‘invasive pannus’ is important since the current biomarkers for RA activity, including erythrocyte sedimentation rate and C reactive protein (CRP) level, often show non-specificity, and there is an unmet need for alternative biomarkers that can adequately reflect disease activity, synovitis severity and therapeutic responses. Proteomics-based approaches have been widely used to obtain biomarkers from various biological materials and diseases.^5^ Previously, Brescia *et al* investigated the cytokines and chemokines secreted from FLSs of patients with juvenile idiopathic arthritis (JIA) and their synovial fluids (SFs) using cytokine antibody arrays.^6^ They identified 29 differentially expressed proteins (DEPs) between JIA and controls in both SFs and FLS culture supernatants, suggesting that these cytokines serve as potential biomarkers for FLS-mediated synovial inflammation. However, this analysis was limited to cytokines and chemokines without providing the global information of the whole secretome of RA-FLSs. Parallel reaction monitoring (PRM) has recently emerged as a promising tool to categorise key proteins among the whole proteome profiled using global proteomic analysis. Accordingly, global proteomic profiling and PRM can be combined to evaluate the RA-FLS-derived secretome and subsequently discover key secreted proteins associated with invasive pannus.

We hypothesised that extracellular and/or secretory molecules from RA-FLSs can reflect the pathological severity of RA, such as synovial proliferation and neovascularisation, and that these molecules may serve as new diagnostic and therapeutic targets. Therefore, we first characterised 843 proteins secreted from RA-FLSs via global proteome profiling of RA-FLS culture supernatants. Using PRM analysis, we then selected the 16 key proteins differentially expressed in the SFs of patients with active RA, including myosin heavy chain 9 (MYH9), which well represented the sonographic severity of synovial proliferation and angiogenesis as well as the level of systemic inflammation of RA. Among the 16 proteins, we particularly focused on MYH9, a component of actin-based cell motility, as a key regulator of ‘invasive pannus’ based on its novelty and the excellent representativeness of pannus pathology on ultrasonography (US). Molecular and functional experiments revealed that the level of MYH9 was increased in RA invasive pannus, and increased MYH9 level promoted RA-FLS migration and invasion in vitro and in vivo. Collectively, our study first provides a comprehensive resource of secretome of RA-FLSs to better understand RA pathogenesis, and suggests that MYH9 is a potential therapeutic target for RA, representing pro-invasive and pro-migratory properties of RA-FLSs.

## Materials and methods

Please see online supplemental materials 1.

10.1136/ard-2022-223625.supp1 Supplementary data

## Results

### Secretome profiling of RA-FLSs

**Figure 1:**
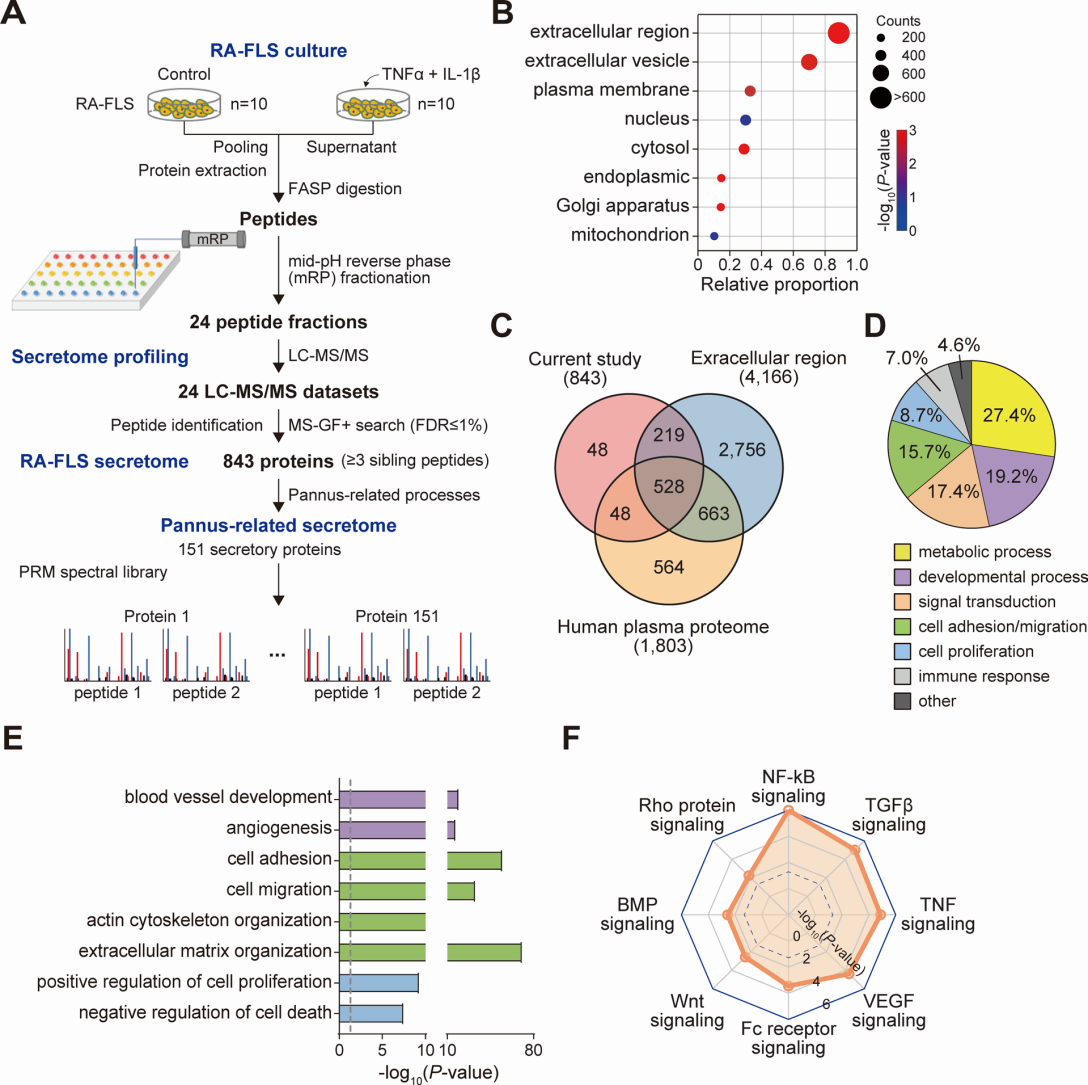
Characteristics of RA-FLS secretome. (A) Overall scheme illustrating experimental steps for RA-FLS culture, secretome profiling, RA-FLS secretome and pannus-related secretome. For pro-inflammatory stimulation, 10 different RA-FLSs were treated with IL-1β (10 ng/mL, n=5), TNFα (10 ng/mL, n=5) or medium alone (n=10) for 24 hours in DMEM containing 1% insulin-transferrin-selenium used as an alternative of FBS. (B) Relative proportions of RA-FLS secretory proteins localised in the indicated Gene Ontology cellular components. The size and colour of dots represent the number of proteins localised in the cellular components and the enrichment p value from DAVID software, respectively. (C) Relationships among RA-FLS secretome, proteins localised in extracellular region and human plasma proteome. (D) Relative proportions of RA-FLS secretory proteins involved in cellular processes related to the indicated processes. (E) GOBPs significantly enriched in RA-FLS secretory proteins. Enrichment significance (p value) was displayed as −log_10_(p value). Bar colours represent the corresponding cellular processes in D. (F) Signalling pathways represented by RA-FLS secretory proteins. Enrichment significance (p value) was displayed as −log_10_(p value). The dotted line indicates the cut-off of p value (0.05). DMEM, dulbecco's modified eagle medium; FASP, filter aided sample preparation; FBS, fetal bovine serum; FLS, fibroblast-like synoviocyte; GOBPs, gene ontology biological processes; IL, interleukin; NF-κB, nuclear factor kappa B; PRM, parallel reaction monitoring; RA, rheumatoid arthritis; TGF, transforming growth factor; TNF, tumour necrosis factor; VEGF, vascular endothelial growth factor.

To evaluate the pro-inflammatory secretome of RA-FLSs, we cultured FLSs isolated from synovial tissues of patients with RA without treatment (control) or with treatment of tumour necrosis factor (TNF)α+interleukin (IL)-1β that mimics inflammatory conditions in RA joints (figure 1A, RA-FLS culture). After pooling the culture supernatants, we fractionated the pooled sample into 24 fractions and performed proteomic profiling of the individual fractions using liquid chromatography-tandem mass spectrometry (LC-MS/MS) analysis (figure 1A, secretome profiling). Using the LC-MS/MS datasets, we identified 843 secretory proteins (online supplemental dataset S1) of RA-FLSs that had more than three unique sibling peptides with false discovery rate <1% via database search using MS-GF+ with UniProt human reference proteome database (figure 1A, RA-FLS secretome). Enrichment analysis of Gene Ontology cellular components showed that the 843 proteins were most significantly enriched with proteins localised in the extracellular region (747 proteins) and extracellular vesicles (EVs; 597 proteins) (figure 1B). Additionally, 94.3% of the 843 proteins overlapped with extracellular proteins or secretory proteins detected in human plasma (figure 1C). Collectively, these data support the validity of our RA-FLS secretome obtained via LC-MS/MS analysis.

10.1136/ard-2022-223625.supp9 Supplementary data

We next examined the cellular processes associated with the 843 proteins released from RA-FLSs by performing enrichment analysis of Gene Ontology Biological Processes (GOBPs) using DAVID software.^7^ The RA-FLS secretome was mainly associated with metabolism (27.4%), developmental process (19.2%), signal transduction (17.4%), cell adhesion/migration (15.7%), cell proliferation (8.7%) and immune response (7.0%) (figure 1D). The metabolism-related RA-FLS secretome most significantly represented glucose metabolism (online supplemental figure S1A), which was consistent with a previous finding.^8^ Notably, the RA-FLS secretome most strongly represented the cellular processes related to pannus formation; overall, 48.5% (409 proteins) of the secretome was associated with pannus-mediated RA pathologies, which included cell migration/invasion (179 proteins), extracellular matrix organisation (177 proteins), cell proliferation/apoptosis (178 proteins), angiogenesis (46 proteins) and pannus-related signalling pathways (21 proteins) (figure 1E, online supplemental figure S1B and table S1). Moreover, the RA-FLS secretome related to signal transduction significantly represented transforming growth factor (TGF)β, bone morphogenic protein (BMP), Wnt,^9^ RhoA and vascular endothelial growth factor (VEGF) signalling,^10^ as well as signal transduction involved in immune responses (nuclear factor kappa B, Fc receptor and TNF signalling) (figure 1F and online supplemental figure S1C). Therefore, the RA-FLS secretome can provide a comprehensive list of RA-FLS secretory factors contributing to pannus formation, which is the pathological hallmark of RA.^11^

### Targeted profiling of pannus-related RA-FLS secretome in SFs

**Figure 2:**
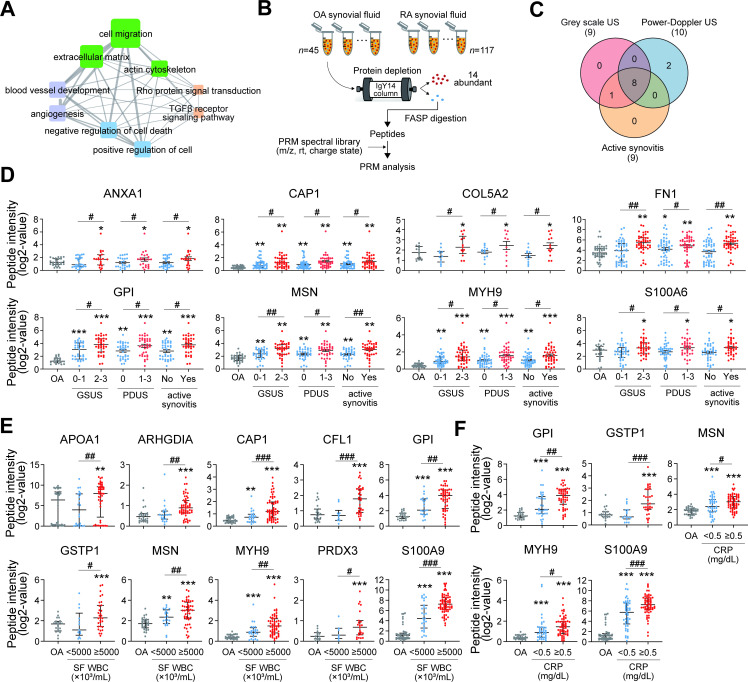
Differential expression of proteins in the pannus-related RA-FLS secretome. (A) GOBP network showing relationships among RA-FLS secretory proteins involved in pannus-related GOBPs. Node side and edge thickness denote the number of proteins involved in each GOBP and the number of overlapping proteins between the connected GOBPs, respectively. Node colours represent the corresponding processes in figure 1D. (B) Overall scheme illustrating experimental steps for PRM analysis. (C) Relationships among the DEPs in the comparisons of GSUS 0 and 1 vs GSUS 2 and 3, PDUS 0 vs PDUS 1–3, and active synovitis versus no active synovitis. (D–F) Differential abundance distributions of eight representative DEPs depending on sonographic severity of synovitis and inflammatory activity of RA. Graphs showing differential expression of the DEPs among OA and the indicated two groups of RA, which were separated by cut-off criteria for sonographic severity (D), SF WBC (5000×10^3^ cells/mL) (E) and blood CRP level (0.5 mg/dL) (F). Top, middle and bottom lines in the dot graphs represent 75th, 50th (median) and 25th percentile values of abundance distributions of the indicated protein. *P<0.05 vs OA group, #p<0.05 vs milder status in RA group. **P<0.01, ***p<0.001, ##p<0.01, ###p<0.001. The p values were determined as described in online supplemental material 1. ANXA1, annexin A1; APOA1, apolipoprotein A1; ARHGDIA, rho GDP dissociation inhibitor alpha; CAP1, cyclase-associated actin cytoskeleton regulatory protein 1; CFL1, cofilin 1; COL5A2, collagen type V alpha 2 chain; CRP, C- reactive protein; DEPs, differentially expressed proteins; FASP, filter aided sample preparation; FLS, fibroblast-like synoviocyte; FN1, fibronectin 1; GOBPs, Gene Ontology biological processes; GPI, glucose-6-phosphate isomerase; GSTP1, glutathione S-transferase pi 1; GSUS, grey-scale ultrasonography; MSN, moesin; MYH9, myosin heavy chain 9; OA, osteoarthritis; PARK7, parkinsonism -associated deglycase; PDUS, power Doppler ultrasonography; PRDX3, peroxiredoxin 3; PRM, parallel reaction monitoring; RA, rheumatoid arthritis; S100A6, S100 calcium binding protein A6; S100A9, S100 calcium binding protein A9; SF, synovial fluid; SOD2, superoxide dismutase.

To further characterise the secretome profiles in RA-FLSs, we performed targeted protein quantification using the PRM method and attempted to determine the clinical correlations. Briefly, we selected 493 of the 843 proteins that were involved in the pannus-related cellular processes (figure 2A). Among them, we selected 151 proteins that contained two or three quantotypic peptides with high quality of MS/MS spectra based on previously described criteria,^12^ and we then constructed a spectral library including the MS/MS spectra of 436 quantotypic peptides for the 151 proteins (see figure 1A, pannus-related secretome). To test the biological relevance of the 151 proteins, we performed PRM analysis of the 151 pannus-related secretory proteins in the SFs, a representative biological fluid in the joints, obtained from patients with RA (n=117) and osteoarthritis (OA) (n=45, non-RA control) via arthrocentesis. Clinical characteristics of patients with RA are summarised in online supplemental table S2. After removing 14 abundant proteins using IgY14 depletion columns, the 151 proteins were quantified in individual SF samples using PRM analysis (figure 2B). The abundances of 277 quantotypic peptides corresponding to 121 proteins were identified in at least one SF sample.

We next investigated which of the 121 pannus-related secretory proteins correlate with the pathological severity of RA, including synovial proliferation and angiogenesis, as simultaneously assessed via musculoskeletal US at the time of SF sampling; US has been widely used for studying RA as it closely reflects the pathological severity of the affected joints in a non-invasive manner.^13^ Therefore, we performed US to compare the abundances of PRM peptides in three groups of patients, including OA (pathological control), mild RA and severe RA.^14^ We identified DEPs that showed increased expression in the severe RA group compared with that in both OA and mild RA groups based on the following severity criteria (figure 2C and online supplemental figure S2A): (1) nine DEPs (annexin A1 (ANXA1), rho GDP dissociation inhibitor alpha (ARHGDIA), cyclase-associated actin cytoskeleton regulatory protein 1 (CAP1), collagen type V alpha 2 chain (COL5A2), fibronectin 1 (FN1), glucose-6-phosphate isomerase (GPI), moesin (MSN), MYH9 and S100 calcium binding protein A6 (S100A6)) in grey-scale US (GSUS) grades of 0 and 1 vs GSUS grades of 2 and 3 (mild vs severe synovial hypertrophy) and (2) 10 DEPs (ANXA1, CAP1, COL5A2, FN1, GPI, MSN, MYH9, parkinsonism-associated deglycase 7 (PARK7), S100A6 and superoxide dismutase 2 (SOD2)) in power Doppler US (PDUS) grade of 0 vs PDUS grades of 1–3. In addition, when active synovitis was defined as GSUS grade of 2 or 3 or PDUS grade of 1–3, nine DEPs (ANXA1, ARHGDIA, CAP1, COL5A2, FN1, GPI, MSN, MYH9 and S100A6) were identified in RA samples with active synovitis compared with those with inactive synovitis (figure 2C and online supplemental figure S2A). Differential expression of eight representative DEPs reflecting synovial hypertrophy, vascularity and active synovitis is shown in figure 2D. We also sought to determine the correlation of the 121 proteins with inflammatory activity of RA. Subsequently, we identified ten DEPs (APOA1, ARHGDIA, CAP1, cofilin 1 (CFL1), GPI, glutathione S-transferase P1 (GSTP1), MSN, MYH9, thioredoxin-dependent peroxide reductase (PRDX3) and S100A9) via the comparison of SF WBC count ≥5000×10^3^ cells/mL versus SF WBC <5000×10^3^ cells/mL and identified five DEPs (GPI, GSTP1, MSN, MYH9 and S100A9) via comparison of blood CRP level ≥0.5 mg/dL vs <0.5 mg/dL (figure 2E, F and online supplemental figure S2B, C).

We ultimately selected 16 DEPs that were strongly correlated with at least one of the aforementioned four measures for RA severity (based on US) and inflammatory activity (based on SF WBC count and blood CRP level). Among the 16 DEPs, APOA1, FN1, GPI and S100A9 have been previously associated with RA pathogenesis as auto-antigens, inflammatory mediators, proliferation inducers or apoptosis inhibitors.^15–18^ Moreover, certain DEPs are known to be produced by RA-FLSs (GPI and S100A9),^19 20^ and other DEPs are involved in proliferation (MSN) and invasiveness of RA-FLSs (ANXA1 and CFL1) and involved in inflammatory cytokine production (CAP1),^10 21–23^ indicating that our PRM analysis results were consistent with previous findings.^10 15–23^ Of note, levels of some DEPs, including GPI, S100A9 and SOD2, showed a positive correlation with RA activity (online supplemental figure S2D, E), assessed by disease activity score 28_ESR_.^24^ In addition, all 16 DEPs have been demonstrated to be detectable in the serum when investigated using the human protein atlas database and/or previously published data, suggesting that they can be studied as potential circulating biomarkers of RA (online supplemental table S3). Collectively, the PRM analysis led to the identification of 16 critical DEPs indicative of ‘invasive pannus’ and ‘inflammatory activity’ of RA, which are thereafter referred to ‘synoviocyte secretome signature’ (SSS)’.

### Identification of MYH9 as a novel candidate indicative of invasive and inflammatory activity of RA-FLSs

**Figure 3:**
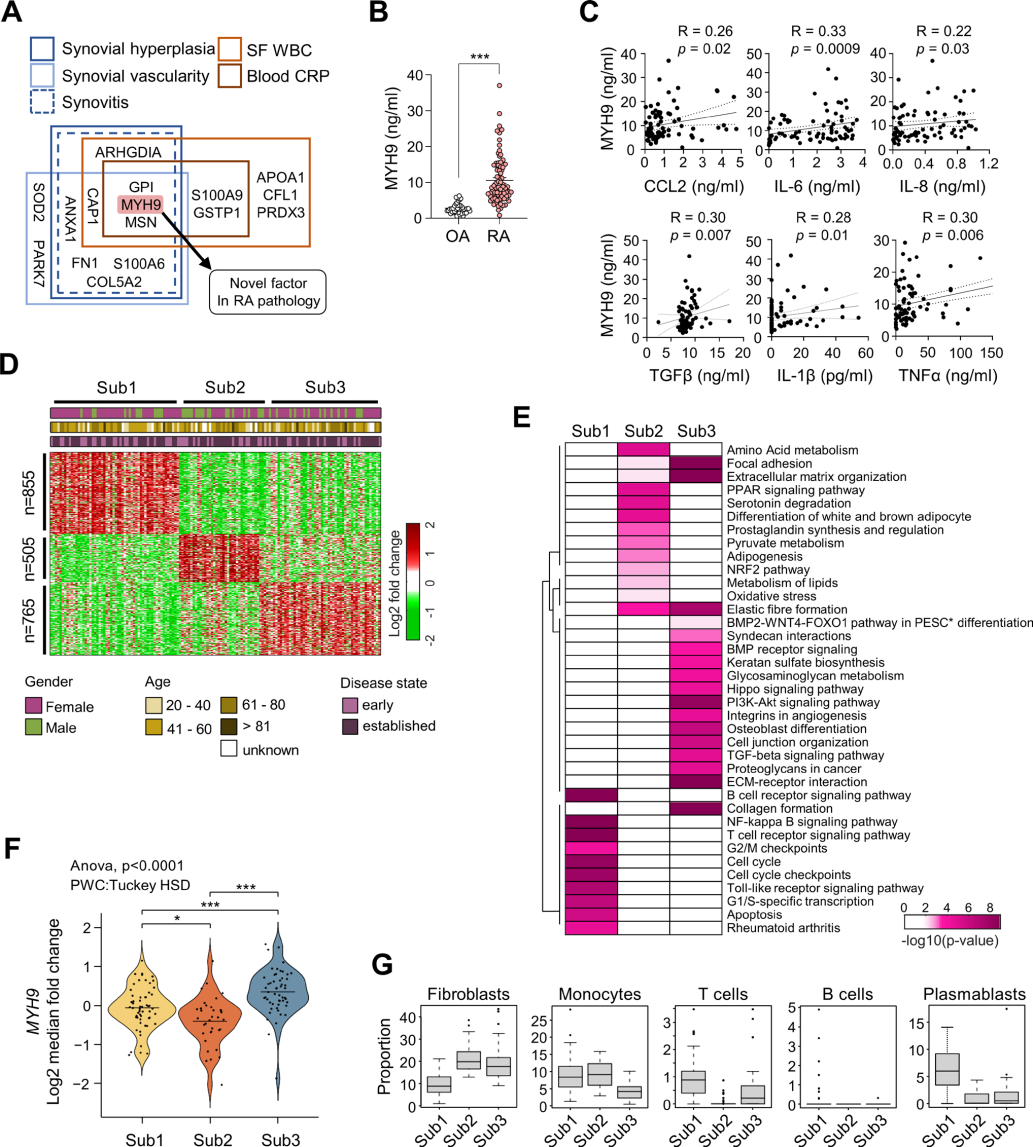
MYH9 as a crucial member of SSS representing the invasiveness of RA-FLSs. (A) A schematic diagram of selection criteria for MYH9 as the most potential constituent of SSS. (B) A quantitative comparison of MYH9 protein levels measured in RA (n=80) and OA (n=40) SFs using ELISA. Bars indicate the mean±SEM. ***P<0.001. The Mann-Whitney U test was used. (C) Correlations of MYH9 level with pro-inflammatory cytokine and chemokine levels in RA SFs (n=80) measured via ELISA. Spearman’s correlation coefficient and the corresponding p value are shown on each plot. (D) Three subtypes (Sub1, Sub2 and Sub3) of patients with RA (n=152) identified by clustering the data obtained from bulk RNA sequencing analysis of their synovial biopsies (GSE89408). The gene numbers included in each cluster are indicated on the left side of the heat map. Sex, age and disease state (early or established) are shown in the top bars. Log_2_-fold-changes of expression levels for each gene in individual samples with respect to the median levels are displayed in the heat map. Colour bar indicates gradient of log_2_-fold-changes. (E) Heat map showing significance of pathways enriched by the signature genes predominantly upregulated in each subtype. The significance is indicated as −log10(p value) where the p value is the enrichment p value from ConsensusPathDB. (F) Violin plot indicating the distribution of *MYH9* expression level in Sub1 to Sub3. The line indicates the average level of *MYH9* expression in each subtype. *P<0.05, ***p<0.001. (G) Box plots showing proportions of the indicated cell types in synovial tissues of patients with RA belonging to Sub1 to Sub3. FLS, fibroblast-like synoviocyte; IL, interleukin; MYH9, myosin heavy chain 9; OA, osteoarthritis; PESC, primary endometrial stromal cell; RA, rheumatoid arthritis; SF, synovial fluid; SSS, synoviocyte secretome signature; TGF, transforming growth factor; TNF, tumour necrosis factor; WBC, white blood cell.

Among the SSS, three proteins including GPI, MYH9 and MSN satisfied all criteria used for selecting the SSS (figure 3A). While GPI and MSN are implicated in RA pathogenesis,^15 21^ the role of MYH9 in RA has been rarely investigated. Thus, we decided to further study this novel target. We first compared MYH9 levels in RA and OA SFs using ELISA and found that RA SFs had considerably higher levels of MYH9 than those in OA SFs (figure 3B). Furthermore, the levels of CCL2, IL-6, IL-8, TGFβ and IL-1β, which are major chemokines and cytokines produced by RA-FLSs, and the level of TNFα, a central activator of RA-FLSs,^25^ were correlated with MYH9 levels in RA SFs (figure 3C). These findings were consistent with our PRM analysis results showing that MYH9 levels increased depending on the severity of synovitis and systemic inflammation (figure 2D, F).

RA is a heterogeneous disease in which diverse immune cells and cellular processes are involved, and its major pathology, although the presence of commonality of pannus formation, can differ depending on disease stage and phenotype.^1^ We questioned whether *MYH9* expression is particularly reflected in a subgroup preferentially exhibiting a pathology of ‘invasive pannus’. Therefore, we first obtained a previously reported bulk RNA-seq dataset generated using synovial tissues of 152 patients with RA,^26^ and identified three major subtypes (Sub1, Sub2 and Sub3) of RA using orthogonal non-negative matrix factorization clustering (figure 3D and online supplemental figure S3A, B) as previously described.^27^ To further characterise the features of each subtype, we attempted to define the major cellular pathways that were represented by the genes predominantly upregulated in each subtype by performing gene set enrichment analysis using ConsensusPathDB.^28^ Sub1 was mainly associated with cellular pathways related to cell proliferation (eg, cell cycle and G2/M checkpoint in figure 3E), Sub2 was associated with pathways related to cell metabolism (eg, pyruvate, amino acid and lipid metabolism in figure 3E) and Sub3 was associated with pathways related to cell migration and invasion (eg, collagen formation, extracellular matrix-receptor interaction and TGFβ/BMP/Hippo signalling in figure 3E), indicating that Sub3 is unique to the invasive pannus. Notably, *MYH9* showed the highest expression level in Sub3 (figure 3F), which is consistent with our findings from the PRM analysis and suggests that MYH9 is related to pro-migratory and pro-invasive pathology to a greater extent than that of its relation with other cellular processes (cell proliferation and metabolism).

We further analysed the cell types enriched in synovial tissues of the three subtypes identified from the bulk RNA-seq data. Briefly, we obtained previously reported single cell RNA-seq data^26^ and identified five cell types, including fibroblasts, monocytes, T cells, B cells and plasmablasts, as well as the genes predominantly upregulated in each cell type (online supplemental figure S3C, D). We then estimated proportions of the five cell types in each synovial tissue of the three subgroups by performing cell-type deconvolution for the corresponding bulk RNA-seq data using CIBERSORTx.^29^ We found that Sub3 contained a predominantly high proportion of fibroblasts, which correspond to RA-FLSs, relative to the proportions of the other four immune cell types (figure 3G). These data, together with increased MYH9 expression in Sub3 (figure 3F), suggest that MYH9 is closely related to fibroblast-dominant pathology in RA synovia, which is in accordance with US analysis results presented in figure 2D.

### MYH9 expression in FLSs and synovia of patients with RA

**Figure 4:**
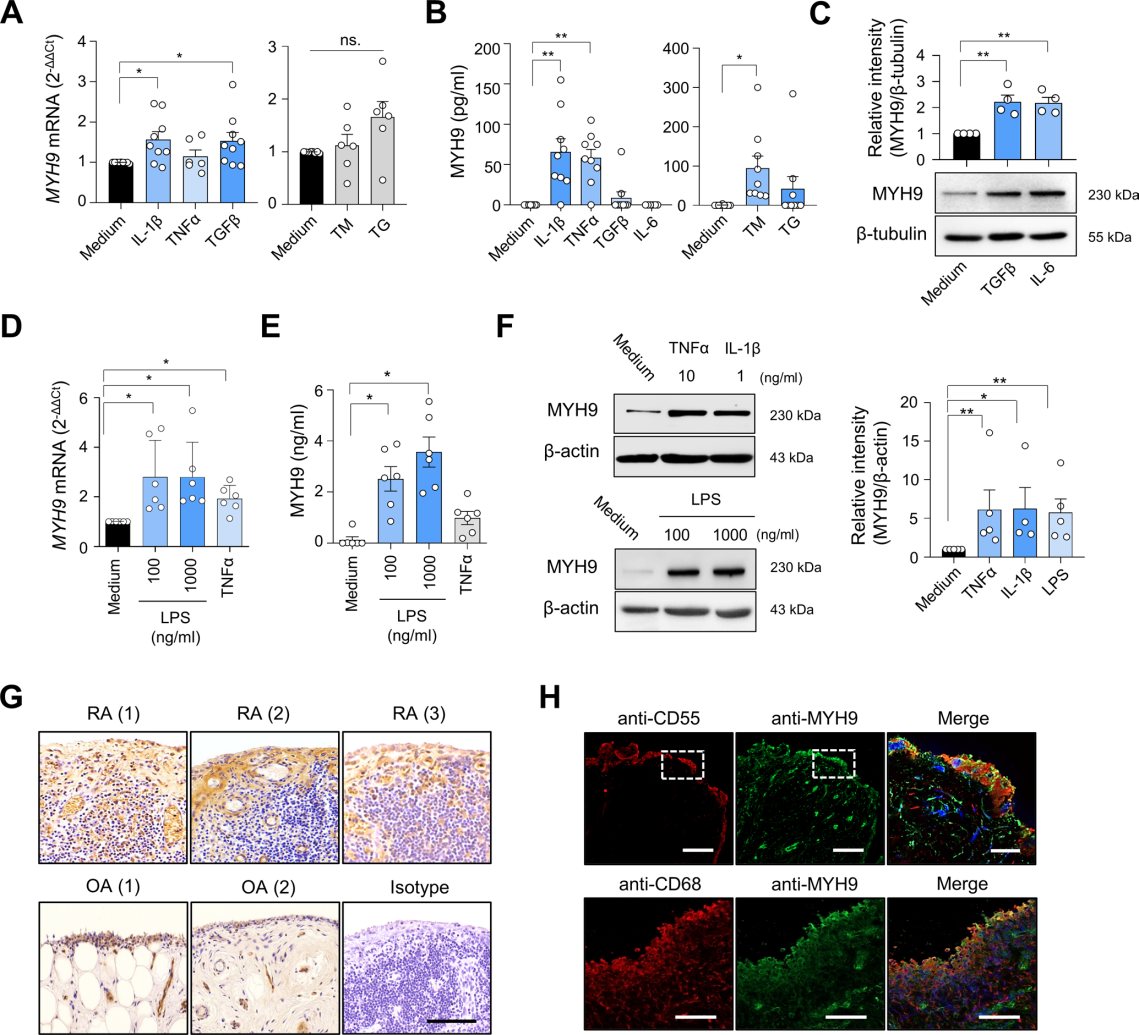
MYH9 expression in RA-FLSs and RA synovium. (A and B) Induction of *MYH9* mRNA expression (A) and secretion (B) by pro-inflammatory cytokines and ER stimuli in RA-FLSs. IL-1β (10 ng/mL), TGFβ (10 ng/mL), TNFα (10 ng/mL), IL-6 (10 ng/mL), TM (10 μg/mL for A; 50 μg/mL for B) and TG (10 μM) were used to treat RA-FLSs (n=6 to 9) for 6 hours (A) or 24 hours (B). *MYH9* mRNA expression in the cells and protein levels in culture supernatants were determined via real-time PCR analysis and ELISA, respectively. (C) Intracellular MYH9 protein levels in RA-FLSs (n=4) treated with TGFβ (1 ng/mL) or IL-6 (1 ng/mL) for 24 hours were determined by immuno-blotting. (D–F) MYH9 induction in human peripheral blood monocytes. CD14^+^ cells (n=6) isolated from blood mononuclear cells using magnetic beads were treated with LPS (100 and 1000 ng/mL), TNFα (10 ng/mL) or IL-1β (1 ng/mL) for 12 hours for mRNA induction or 24 hours for analysis of protein expression. *MYH9* mRNA expression in the cells was determined by qRT-PCR (D). Levels of MYH9 in the culture supernatants and in the cells were determined by ELISA (E) and western blot analysis (F), respectively. (G) Immunohistochemical staining of MYH9 in RA and OA synovial tissues. An isotype control antibody was used as a control. Scale bar, 100 μm. (H) Immunofluorescence staining of RA synovium using anti-MYH9 Ab (green), anti-CD55 Ab for RA-FLSs (red) and anti-CD68 Ab for macrophages (red). Co-localisation of MYH9 with CD55 (top panel) or with CD68 (bottom panel) is visualised in yellow in the merged images. Nuclei were stained with DAPI (blue). The rectangular area with a white dashed line was magnified to the merged image. Scale bars, 100 μm (for a magnified image, 20 μm). Data show representatives of more than three independent experiments or the mean±SEM. *P<0.05 and **p<0.01. The p values were determined via the Mann-Whitney U test. DAPI, 4′,6-diamidino-2-phenylindole; FLS, fibroblast-like synoviocyte; IL, interleukin; LPS, lipopolysaccharide; MYH9, myosin heavy chain 9; ns, not significant; OA, osteoarthritis; RA, rheumatoid arthritis; TG, thapsigargin; TGF, transforming growth factor; TM, tunicamycin; TNF, tumour necrosis factor.

Based on PRM and cell-type deconvolution analysis, we validated whether MYH9 is actually expressed in RA-FLSs, contributing to FLS migration and invasion. As expected, *MYH9* mRNA was expressed in RA-FLSs based on qPCR analysis and its expression was increased on stimulation with pro-inflammatory cytokines, including IL-1β and TGFβ (figure 4A). MYH9 protein was similarly secreted by RA-FLSs stimulated with IL-1β and TNFα, but not with TGFβ, as measured using ELISA (figure 4B). We previously demonstrated that RA-FLSs are heavily exposed to endoplasmic reticulum (ER) stimuli, such as hypoxic and pro-inflammatory stimuli.^30^ Notably, treatment of RA-FLSs with tunicamycin, an ER stressor,^31^ substantially increased MYH9 secretion without affecting *MYH9* mRNA levels (figure 4A, B); another ER stressor, thapsigargin, failed to induce MYH9 secretion (figure 4B). Interestingly, TGFβ and IL-6 treatment did not instigate (extracellular) MYH9 secretion from RA-FLSs (figure 4B), but it did substantially upregulate (intracellular) MYH9 expression in the RA-FLSs as determined by western blot analysis (figure 4C). These data suggest that MYH9 secretion by RA-FLSs can be induced by pro-inflammatory cytokine IL-1β and TNFα as well as tunicamyin and that it does not seems to be necessarily correlated with its expression levels in the cells.

RA synoviocytes are composed of not only FLSs, but also macrophage-like synoviocytes (MLSs).^32^ MLSs and recruited monocytes from blood are required for perpetuation of chronic inflammation in RA joints.^33^ We found that both *MYH9* mRNA expression and MYH9 protein secretion were substantially increased in human blood-derived monocytes after treatment with LPS (figure 4D, E). TNFα also modestly increased *MYH9* mRNA expression in the monocytes but failed to elevate MYH9 secretion markedly like LPS (figure 4D, E). Of note, MYH9 secretion by cultured monocytes was considerably higher than that by RA-FLSs (see ELISA results in figure 4B vs E). Moreover, stimulation of the peripheral monocytes with LPS, TNFα and IL-1β significantly increased (intracellular) MYH9 expression as determined by western blot analysis (figure 4F). These data suggest that TLR4 ligation and pro-inflammatory cytokine TNFα and IL-1β can increase MYH9 secretion and/or expression in human monocytes.

Immunohistochemistry revealed that MYH9 expression was higher in RA synovia than in OA synovia, particularly in the lining layer and sublining leucocytes (figure 4G). Immunofluorescence staining similarly demonstrated that MYH9-expressing cells were considerably co-localised with anti-CD55^+^ and anti-CD68^+^ cells, confirming that FLSs and MLSs represented the major MYH9-expressing cells in the RA synovium (figure 4H).

Collectively, MYH9 is expressed in FLSs and MLSs in the RA synovium and can be secreted (or excreted) from these cells on stimulation with pro-inflammatory cytokines, toll-like receptor agonist (LPS) or ER stress.

### Essential role of MYH9 in migration and invasion of RA-FLSs

**Figure 5:**
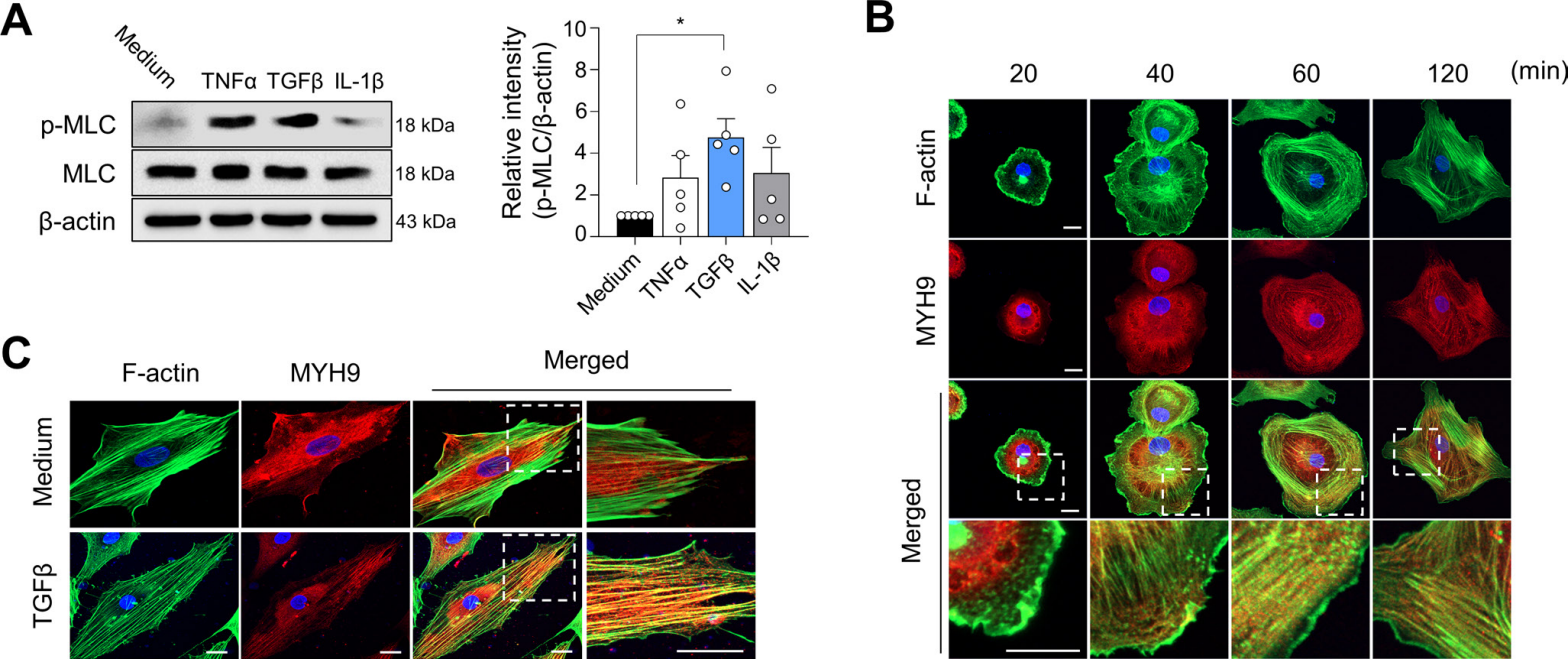
MYH9 activation by TGFβ and its co-localisation with F-actin in RA-FLSs. (A) Increase in levels of MLC phosphorylation (at Thr18/Ser19) in RA-FLSs stimulated with TNFα (10 ng/mL), TGFβ (10 ng/mL) and IL-1β (1 ng/mL) for 15 min as determined via western blot analysis. Data show representatives of more than three independent experiments or the as mean±SEM. *P<0.05. The p value was calculated using Mann-Whitney U test. (B) Cell-spreading assay of RA-FLSs. RA-FLSs were seeded onto cover glasses coated with fibronectin, incubated to attach to the glasses for the indicated times and then fixed with paraformaldehyde. The cells undergoing spreading were double-immunostained using phalloidin for F-actin (green) and anti-MYH9 Ab (red). (C) Co-localisation of MYH9 and F-actin in RA-FLSs stimulated with TGFβ. RA-FLSs (5×10^3^ cells per well) were incubated overnight onto the 8-well chamber slides (with no fibronectin), stimulated by medium alone or TGFβ (10 ng/mL) for 6 hours, and then stained with phalloidin (green) and anti-MYH9 Ab (red). The rectangular areas with a white dashed line in each merged image were magnified and shown in the adjacent panel. The confocal images are representatives of more than three experiments. FLS, fibroblast-like synoviocyte; IL, interleukin; MLC, myosin light chain; MYH9, myosin heavy chain 9; RA, rheumatoid arthritis; TGF, transforming growth factor; TNF, tumour necrosis factor.

*MYH9* encodes the heavy chain of non-muscle myosin IIA.^34^ Non-muscle myosin IIA, which is composed of MYH9 and MLC, is an indispensable factor in cell adhesion and migration.^34^ As enhanced migration and invasion of FLSs represent one of the critical pathologies of RA, we investigated the role of MYH9 in these cellular processes. We found that MLC phosphorylation, by which non-muscle myosin can transform into an active form,^34^ was significantly increased in RA-FLSs treated with TNFα and TGFβ, suggesting that pro-inflammatory cytokine stimulation enhances the MYH9-dependent activity of RA-FLSs (figure 5A). It is well known that activated non-muscle myosin IIA binds to F-actin through its head domain.^34^ In cell-spreading assay (figure 5B), we found that 20 min after attachment of suspended RA-FLSs to cover glasses coated with fibronectin, MYH9 was diffusely distributed in the cytoplasm while F-actin was predominantly localised in the cell edges, where actin cytoskeleton undergoes extensive remodelling, as determined by double immuno-staining of F-actin and MYH9. Over time, MYH9 was detected primarily in the cell edges and co-localised with F-actin 60 min and 120 min after the incubation of RA-FLSs onto fibronectin-coated glasses. Moreover, after a stable attachment of RA-FLSs overnight, most MYH9 was diffusely redistributed in the cytoplasm, but with TGFβ stimulation, some MYH9 molecules were found in the cell edges and co-localised with F-actin in the actin cytoskeleton (figure 5C). Together, these results support the earlier reports that MYH9 activity can be induced by TGFβ and binds to F-actin in tumour cells.^35 36^

**Figure 6:**
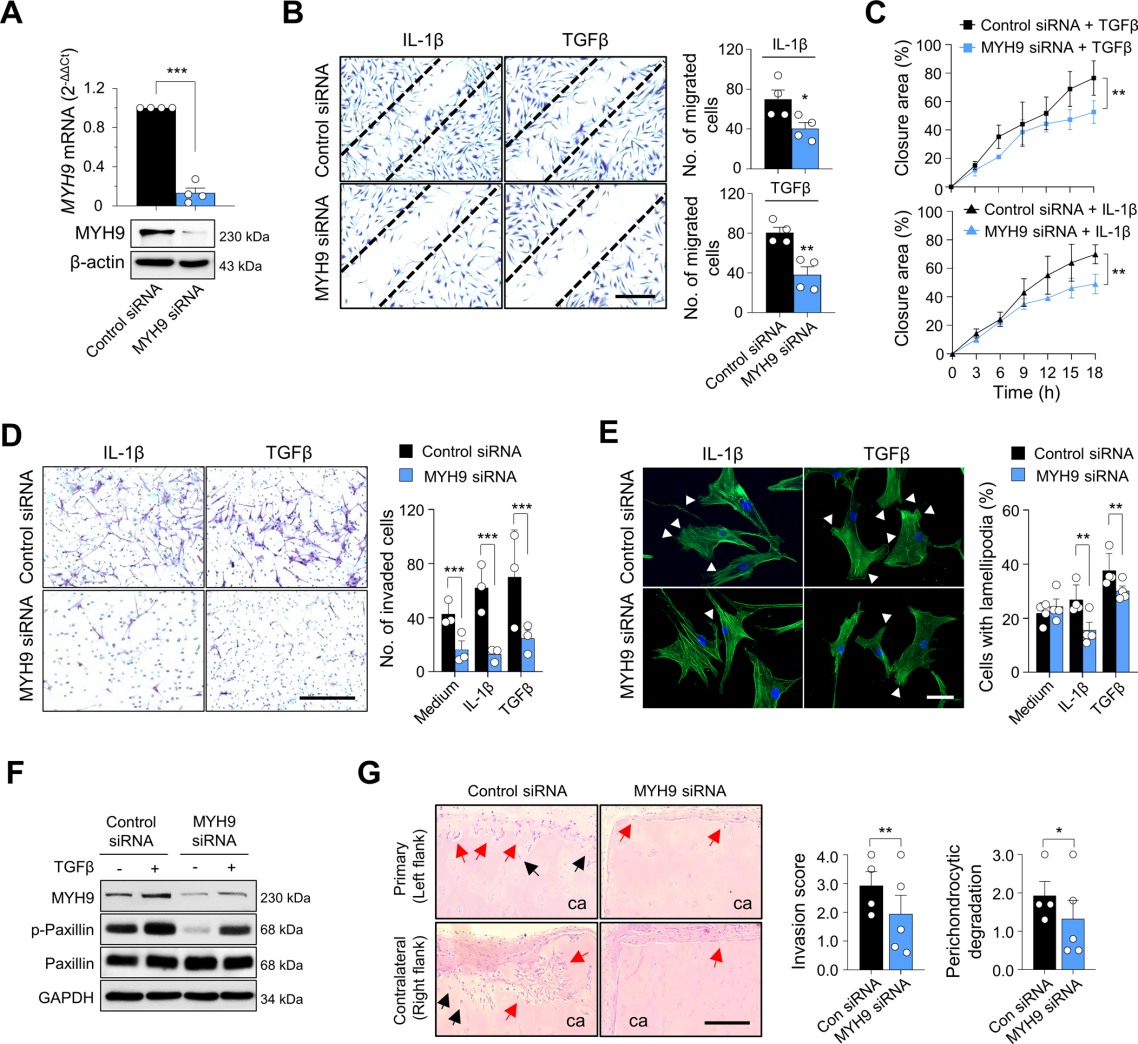
Effect of MYH9 on migration and invasion of RA-FLSs. (A) Reduction of *MYH9* expression in RA-FLSs via *MYH9* siRNA transfection. RA-FLSs (n=4) were transfected with 50 nM of *MYH9* siRNA for 48 hours. *MYH9* mRNA expression (top panel) and protein levels (bottom panel) were determined via real-time PCR and western blot analysis, respectively. (B–D) Decrease in migration and invasion of RA-FLSs via knockdown of *MYH9*. RA-FLSs (n=3) were transfected with *MYH9* siRNA (50 nM) for 24 hours. The cells were then wounded using pipette tips (B and C) or loaded onto a Matrigel chamber (D). IL-1β (1 ng/mL) and TGFβ (10 ng/mL) in DMEM containing 1% FBS were added to wounded cells (B and C) or to lower chambers of Matrigel-coated Transwell (D) for 12 hours. Migrated (B) and invaded cells (D) were stained using crystal violet solution and then manually counted. The wounding closure areas were also monitored in real time (C). Scale bar, 200 μm. (E) Lamellipodium formation suppression by *MYH9* siRNA in RA-FLSs. RA-FLSs (n=4) were transfected with *MYH9* siRNA (50 nM) in DMEM supplemented with 10% FBS for 24 hours and subsequently incubated with IL-1β (1 ng/mL) or TGFβ (10 ng/mL) in DMEM containing 1% FBS for 24 hours. The cells were then stained with Alexa Fluor 488-conjugated phalloidin (green) for F-actin visualisation. Nuclei were stained with DAPI (blue). White triangles indicate lamellipodia. Scale bar, 20 μm. (F) MYH9-dependent regulation of focal adhesions in RA-FLSs. RA-FLSs (n=4) were transfected with *MYH9* siRNA (50 nM) for 48 hours and then incubated in the absence or presence of TGFβ (10 ng/mL) for 12 hours. Protein levels of MYH9, total paxillin and phosphorylated paxillin at Y118 site (p-paxillin) were measured via western blot analysis. The data are representatives of more than three experiments. (G) Suppression of RA-FLS invasion into cartilages via *MYH9* knockdown in a humanised synovitis model. RA-FLSs were transfected with control siRNA (50 nM) (n=4) or *MYH9* siRNA (50 nM) (n=5) for 24 hours. Human cartilages were implanted into the left flank (primary, ipsilateral) with RA-FLSs for 60 days in SCID mice. In the right flank (contralateral), the cartilages of the same size were implanted without RA-FLSs. At day 60, the implants were harvested and subjected to H&E staining. The red and black arrows indicate invaded regions and perichondrocytic degradation, respectively. Scale bar, 100 μm. Data are presented as the mean±SEM. *P<0.05, **p<0.01 and ***p<0.001 vs control siRNA-treated groups. The p values were calculated using Mann-Whitney U test (A, B, D, E and G) or two-way analysis of variance followed by Sidak’s post-test (C). DMEM, dulbecco's modified eagle medium; FBS, fetal bovine serum; FLS, fibroblast-like synoviocyte; MYH9, myosin heavy chain 9; RA, rheumatoid arthritis; SCID, severe combined immunodeficiency; siRNA, small interfering RNA; TGF, transforming growth factor.

To determine the effects of MYH9 on migration and invasion of RA-FLSs, *MYH9* was knocked down using siRNAs (figure 6A). RA-FLS migration (figure 6B), real-time wound migration (figure 6C, online supplemental figure S4A and online supplemental movies S1A–D), and invasion (figure 6D) were all significantly reduced after *MYH9* knockdown; meanwhile, the *MYH9* knockdown did not affect cell viability (online supplemental figure S5A). Moreover, the number of RA-FLSs containing lamellipodia, which are well-known subcellular structures representing actin reorganisation of migrating cells,^37^ was correspondingly decreased via *MYH9* knockdown (figure 6E). In parallel, MYH9 deficiency significantly impaired paxillin phosphorylation (figure 6F), which is an essential step of focal adhesion and is indispensable for cell migration dynamics.^38^ Taken together, these observations, along with earlier reports,^39–41^ suggest that MYH9 activity and expression can be increased by TGFβ stimulation, which induces the phosphorylation of paxillin as well as MYH9 binding to F-actin, ultimately promoting adhesion, migration and invasion of RA-FLSs (online supplemental figure S6).

10.1136/ard-2022-223625.supp2 Supplementary video

10.1136/ard-2022-223625.supp3 Supplementary video

10.1136/ard-2022-223625.supp4 Supplementary video

10.1136/ard-2022-223625.supp5 Supplementary video

To further investigate the role of MYH9 in RA-FLS aggressiveness in vivo, we developed an SCID mouse-xenograft model, which is a humanised synovitis model in which human cartilages are implanted with RA-FLSs in the left flank of SCID mice and without RA-FLSs in the right flank of the same mice. The cartilages implanted with *MYH9*-deficient RA-FLSs showed significantly decreased levels of destruction and degradation in both ipsilateral (left flank) and contralateral (right flank) sides (figure 6G), indicating that *MYH9* knockdown suppresses the local invasiveness of RA-FLSs and may retard distant migration of RA-FLSs from the affected joint to the unaffected cartilages.

**Figure 7:**
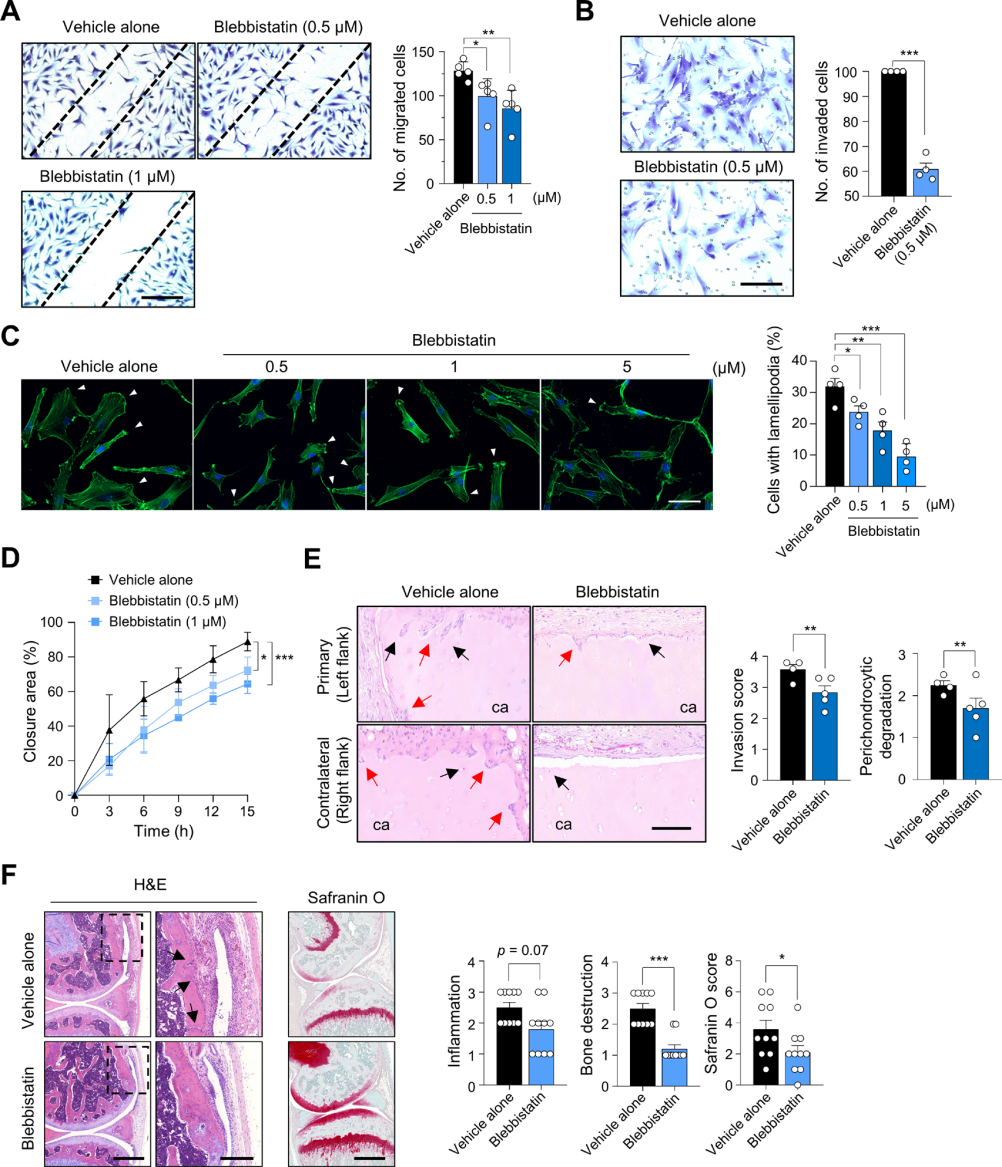
Reduction of migration and invasion of RA-FLSs by blebbistatin, a specific inhibitor of non-muscle myosin II. (A and B) Repression of wound migration and invasion of RA-FLSs by blebbistatin. RA-FLSs were wounded (A, n=5) using pipette tips or loaded onto upper chambers of Matrigel-coated Transwell (B, n=4). They were then treated with vehicle alone (containing dimethyl sulfoxide) or blebbistatin at the indicated concentrations. After 12 hours of incubation, the cells were stained with crystal violet solution and the migrating cells were manually counted. (C) Decrease in lamellipodium formation in RA-FLSs by blebbistatin. RA-FLSs (n=4) were cultured in DMEM supplemented with 5% FBS in the absence or presence of blebbistatin at the indicated concentrations for 12 hours. The cells were stained with Alexa Fluor 488-conjugated phalloidin (green) to visualise F-actin. Nuclei were stained with DAPI (blue). The number of lamellipodium-containing cells was manually counted. Scale bar, 20 μm. (D) Wounding migration of RA-FLSs (n=3) was evaluated in the same manner as shown in (A) and monitored in real-time for 16 hours. (E) Blebbistatin-mediated repression of RA-FLS invasion into cartilages in a humanised synovitis model. Human cartilages were implanted in the left flank (primary, ipsilateral) with RA-FLSs for 60 d in SCID mice. In the right flank (contralateral), cartilages of the same size were implanted without RA-FLSs. The mice were injected with vehicle alone (n=5) or blebbistatin (n=5) at a dose of 10 mg/kg twice a week for 60 days. At day 60, the implants were harvested and subjected to H&E staining. The red arrows and black arrows indicate invaded regions and perichondrocytic degradation, respectively. Scale bar, 100 μm. The bar graphs indicate severity of cartilage invasion and degradation. (F) Amelioration of methylated bovine serum albumin (BSA) plus IL-1β-induced arthritis by blebbistatin. The mice were injected intraperitoneally with blebbistatin (10 mg/kg, n=10) or vehicle alone (n=10) daily from 4 days after arthritis induction. The images in the left panel are representative of H&E and safranin O-stained joints. The rectangular areas with a black dashed line in each H&E-stained image were magnified and shown in the adjacent panel. Scale bars, 500 μm (for magnified images, 200 μm). Black arrows indicate bone destruction. The bar graphs in the right panel are presented as mean±SEM. *P<0.05, **p<0.01, and ***p<0.001 vs vehicle-treated groups. The p values were determined via Mann-Whitney U test (A, B, C, E and F) or two-way analysis of variance followed by Sidak’s post-test (D). DMEM, Dulbecco's Modified Eagle Medium; FBS, fetal bovine serum; FLS, fibroblast-like synoviocyte; MYH9, myosin heavy chain 9; RA, rheumatoid arthritis; SCID, severe combined immunodeficiency.

The in vitro and in vivo findings using siRNAs strongly suggest that MYH9 represents an excellent target to regulate FLS migration and invasion. Therefore, we tested whether blebbistatin, a specific MYH9 inhibitor that hinders the activity of non-muscle myosin II,^42^ can reverse cartilage destruction in vivo and suppress the pro-migratory and pro-invasive phenotype of RA-FLSs in vitro. The migration, invasion, lamellipodium formation and real-time wound migration of RA-FLSs were all substantially suppressed via blebbistatin treatment without affecting cell viability in vitro (figure 7A–D, online supplemental figure S4B and S5B and online supplemental movies S1E–G), which is consistent with the findings of the knockdown experiment using *MYH9* siRNAs.

10.1136/ard-2022-223625.supp6 Supplementary video

10.1136/ard-2022-223625.supp7 Supplementary video

10.1136/ard-2022-223625.supp8 Supplementary video

Moreover, in the in vivo humanised synovitis model using SCID mice, intraperitoneal injection of blebbistatin (10 mg/kg twice a week) significantly reduced RA-FLS-mediated cartilage degradation in both ipsilateral and contralateral sides (figure 7E). These results indicate that blebbistatin inhibited the local invasiveness of RA-FLSs in the ipsilateral side, and it prevented their migration to the contralateral side, where RA-FLSs were not originally implanted. To further confirm these findings in more complex models of arthritis, where diverse immune cells, in addition to FLSs, are involved in disease progression, we introduced two different mouse models of chronic inflammatory arthritis, including methylated bovine serum albumin (BSA)/IL-1β-induced arthritis and collagen-induced arthritis. The result showed that intraperitoneal injection of blebbistatin (10 mg/kg daily) remarkably reduced bone and cartilage destruction in mice with BSA/IL-1β-induced arthritis, although its effect on inflammatory cell infiltration in the affected joints was modest (figure 7F); most mice tolerated even repetitive treatment of blebbistatin, and there were no apparent adverse effects or toxicity, including thrombocytopenia (data not shown). The severity of collagen-induced arthritis also was significantly lower in mice treated with blebbistatin (10 mg/kg twice a week) than in mice with vehicle alone (online supplemental figure S7A, B). In parallel, inflammatory cell infiltration and bone/cartilage destruction in these mice were substantially diminished by intraperitoneal injection of blebbistatin (online supplemental figure S7C). These data demonstrate that blebbistatin treatment is effective in ameliorating joint destruction by diverse immune/inflammatory cells as well as in retarding joint destruction and disease spread mediated predominantly by RA-FLSs.

Collectively, these data confirm that MYH9 is essential for the migration and invasion of RA-FLSs and demonstrated that MYH9 inhibition by blebbistatin successfully represses local invasion and cartilage degradation mediated by RA-FLSs in vitro and in vivo.

## Discussion

The abnormal activation and proliferation of FLSs are considered a pathological hallmark of RA. In the RA joints, proteins secreted from FLSs may critically induce the development of such pathology; however, they have never been globally and systematically explored. To address these gaps in knowledge, we determined the secretome profile of RA-FLSs, the major cell type comprising the invasive pannus, via LC-MS/MS analysis. We then attempted to evaluate the invasive pannus-related secretome using PRM analysis; we selected the 16 proteins on the basis of sonographic severity of synovial proliferation and angiogenesis as well as the level of systemic inflammation of RA, after which they were called ‘SSS’. Particularly, MYH9, one of the previously unidentified SSS in RA, showed a strong relationship with inflammatory activity in the SF and strong relation with fibroblastic activity in the transcriptome profile of RA synovia. MYH9 expression was elevated in RA-FLSs and RA synovium and it was induced by pro-inflammatory cytokines (eg, IL-1, TNFα, TGFβ and IL-6), TLR ligation and ER stimuli. Finally, we functionally validated that MYH9 was co-localised with F-actin and promoted the migration and invasion of RA-FLSs in vitro and in vivo. Collectively, this study may represent substantial advances in our understanding of chronic inflammatory diseases in that it first introduces the concept of secretome to inflammatory arthritis, provides a list of ‘SSS’ related to pannus formation and suggest potential new targets, including MYH9, for the evaluation and management of human diseases where fibroblasts play a pivotal role (online supplemental figure S8).

Functional enrichment analysis revealed that 27.4% of the RA-FLS-derived secretome was involved in metabolic processes, and glycolysis-related proteins were the most abundant, including GPI, triose phosphate isomerase and aldolase A. The RA-FLS-derived secretome also included pyruvate kinases and GAPDH, which are rate-limiting enzymes of glycolysis. These proteins are upregulated under hypoxic conditions in RA synovial tissues.^43^ In addition, 19.2% of the RA-FLS-derived secretome was involved in developmental processes. RA-FLSs show characteristics of dedifferentiated cells resembling mesenchymal stem cells.^44^ Therefore, our secretome profile may reflect such cellular processes as embryonic synovial development and dedifferentiation of RA-FLSs. Notably, the RA-FLS-derived secretome highly represented angiogenesis, cell adhesion/migration and proliferation, which are closely related to the pathology of invasive pannus; 48.5% (409 proteins) of the secretome was associated with pannus-driven pathologies. Collectively, the global profiling of RA-FLS secretome further confirmed the essential role of RA-FLSs in RA pathogenesis.

Our secretome data also provide novel perspectives on secreted proteins from RA-FLSs. Specifically, we sorted 16 proteins of the SSS characterising the invasive pannus using PRM analysis. Since the 16 proteins covered several autoantigens and were located in an extracellular region, the SSS may function as autoantigens: FN1 as an autoantigen and a disease-facilitating factor in a citrullinated form^16^; GPI as one of the autoantigens in RA^15^ and COL5A2 as a potential inducer of autoimmune responses.^45^ The SSS also included diverse damage-associated molecular patterns (DAMPs) derived from stressed, damaged or dying cells (eg, FN1, S100A9, APOA1 and PARK7),^46–48^ which may contribute to the persistence of joint inflammation. Notably, MYH9 plays a role in both autoantigen cross-presentation of dendritic cells^49^ and inflammatory responses driven by immune complex of MYH9 and natural IgM.^50^ Collectively, previous studies^15 16 45–50^ suggest that the SSS may be involved in facilitating autoimmune responses as a self-antigen as well as in perpetuating chronic inflammation as a DAMP, which needs to be further studied.

Among the 16 proteins of the SSS, we investigated MYH9 as a key regulator of the invasive pannus based on its novelty and correlation with pathology related to US. MYH9 is a key factor in actin-based cell motility. Especially, the migration and extravasation of leucocytes, including neutrophils and T lymphocytes, are largely dependent on MYH9.^34^ MYH9 function is also crucial to epithelial-to-mesenchymal transition induced by TGFβ.^51^ MYH9 involvement in the enhanced motility of cancer cells has been reported in certain types of cancer.^52^ Here, we demonstrated that IL-1β-induced and TGFβ-induced RA-FLS migration and invasion were severely suppressed on *MYH9* knockdown. Moreover, *MYH9* depletion induced a marked decrease in invasion and cartilage destruction mediated by RA-FLSs in the in vivo humanised synovitis model. Overall, our data, together with previous findings,^34 51 52^ suggest that MYH9 is a key regulator of tumour-like invasion and migration of RA-FLSs. Similar to MYH9, future studies should investigate the role of other SSS proteins whose functions are not fully understood in RA, including S100A6, ARHGDIA, GSTP1 and PRDX3.

Notably, non-muscle myosin IIA promotes the release of EVs.^53^ EVs are crucial in cell-cell communication^54^ and have been implicated in the pathogenesis of RA.^55^ The number of EVs is significantly increased in patients with RA and they can activate RA-FLSs to induce pro-inflammatory mediators and cartilage-degrading enzymes.^55^ In our study, approximately 70% of the secretome of RA-FLSs represented EV-associated proteins, which implies that a substantial portion of the SSS can be transferred to neighbouring FLSs and other types of immune cells via EVs in RA joints and may explain why MYH9 secretion is not always correlated with its expression level in RA-FLSs (figure 4A–C). Moreover, pro-inflammatory stimuli and TLR ligation enhanced MYH9 release and induced MLC phosphorylation that presumably further accelerates the release of EVs.^53^ Given the crucial role of MYH9 in the invasiveness of RA-FLSs, these EVs harbouring MYH9, which are possibly secreted from relatively more aggressive FLSs, can be delivered to other FLSs and make them more aggressive, which may represent a potential mechanism underlying the spreading of transformed phenotype of RA-FLSs (online supplemental figure S6).

Therapeutic approaches targeting RA-FLSs have been developed to treat RA; however, there are no clinically approved drugs available at present.^56^ Suppression of RA-FLSs using monoclonal antibodies against cadherin-11 has failed to show sufficient therapeutic efficacy in a phase II clinical trial.^56^ Seliciclib, a cyclin-dependent kinase inhibitor, has been reported to suppress proliferation of RA-FLSs; however, it currently only satisfies the toxicity requirements in a phase I clinical study.^56^ Targeting non-muscle myosin to regulate RA-FLSs has never been attempted in RA treatment. Here, we showed that blebbistatin, an inhibitor of non-muscle myosin II, remarkably suppressed the aggressiveness of RA-FLSs in vitro and in vivo, thereby reducing cartilage destruction in an SCID mouse-xenograft (RA-FLSs) model. Blebbistatin treatment is also effective in retarding joint destruction by diverse immune cells in more complex mouse models of chronic arthritis, including methylated BSA/IL-1β-induced arthritis and collagen-induced arthritis. These findings suggest that the development of myosin-targeted therapeutics while focusing on RA-FLS-driven pathologies can provide an alternative strategy for RA treatment. Given the modest effect of blebbistatin on cartilage destruction in SCID mouse model, further studies, including dose-dependent experiments using MYH9 inhibitors, will be required to clarity this issue.

In conclusion, our study provides a comprehensive resource of an RA-FLS-derived secretome that can be applied in various studies to help discover novel regulators of pathological processes mediated by proteins secreted by RA-FLSs. Moreover, PRM analysis of the RA-FLS-derived secretome revealed 16 secretory proteins comprising the SSS which represented the pathology of the invasive pannus. Similar PRM analyses should be applied to identify the key secretory proteins responsible for other processes underlying RA pathogenesis, including altered metabolism, abnormal proliferation and de-differentiation of RA-FLSs since it successfully sorted the invasive pannus-related SSS. Finally, we propose MYH9 as a promising target for retarding abnormal migration and invasion of RA-FLSs and also suggest potential therapeutic candidates of the SSS, for which detailed functional experiments can be designed.

## Data Availability

Data are available in a public, open access repository. Data are available on reasonable request. The proteomics data and PRM analysis data were deposited to the ProteomeXchange with accession ID: PXD041077. Other data are available on reasonable request.
