# Supplementary material for: Identification of MYH9 as a key regulator for synoviocyte migration and invasion through secretome profiling
Source: Ann Rheum Dis. 2023 May 15;82(8):1035–48. doi: 10.1136/ard-2022-223625 (PMC10359537; doi:10.1136/ard-2022-223625)
Supplement: Supplementary data [file ard-2022-223625supp001.pdf]

Supplemental Materials

Identification of MYH9 as a key regulator for synoviocyte migration and invasion through secretome profiling

Saseong Lee[1,*],Eunbyeol Choi[1,2,*], Sehyun Chae[3,*], Jung Hee Koh[1,4], Yoolim Choi[5], Jung Gon Kim[1,6], Seung-Ah Yoo[1,7], Daehee Hwang[5], and Wan-Uk Kim[1,4]

[1]Center for Integrative Rheumatoid Transcriptomics and Dynamics, The Catholic University of Korea, Seoul, 06591, Korea.
[2]Department of Biomedicine & Health Sciences, College of Medicine, The Catholic University of Korea
[3]Neurovascular Unit Research Group, Korean Brain Research Institute, Daegu, 41062, Korea
[4]Department of Internal Medicine, The Catholic University of Korea, Seoul, 06591, Korea.
[5]School of Biological Sciences, Seoul National University, Seoul, 08826, Korea.
[6]Department of Internal Medicine, Ilsan Paik Hospital, Inje University, College of Medicine, Goyang, 10380, Republic of Korea.
[7]Department of Medical Life Sciences, The Catholic University of Korea, Seoul, 06591, Korea.
*These authors contributed equally.

Correspondence: Dr. Wan-Uk Kim, Division of Rheumatology, Department of Internal Medicine, The Catholic University of Korea, School of Medicine, 222, banpo-daero, seocho-gu Seoul, 06591, Korea. Phone: 82.2.3147.8762; E-mail: wan725@catholic.ac.kr, Dr. Daehee Hwang, School of Biological Sciences, Seoul National University, 1, guanak-ro, guanak-gu, Seoul, 08826, Korea. Phone: 82.2.880.2859; Email: daehee@snu.ac.kr, or Dr. Seung-Ah Yoo, The Catholic University of Korea, School of Medicine, 222, banpo-daero, seocho-gu Seoul, 06591, Korea. Phone: 82.2.3147.8410; E-mail: youcap78@hanmail.net.

This PDF file includes:

Supplemental Methods
Supplemental References
Figures S1 to S8
Tables S1 to S3
Legend for Movie S1
Legend for Dataset S1

Other supporting materials for this manuscript include the following:

Movie S1
Dataset S1

Lee S, *et al. Ann Rheum Dis* 2023; 82:1035–1048. doi: 10.1136/ard-2022-223625

Supplemental Methods

Study population and SF sampling

Patients with RA (n = 180) and OA (n = 82) for SF sampling were enrolled from the Center for Integrative Rheumatoid Transcriptomics and Dynamics cohort consisting of patient groups at Seoul St. Mary's Hospital, Seoul, Korea. All patients with RA participating in this study satisfied the European League Against Rheumatism and the 2010 American College of Rheumatology criteria.[1] As controls, patients with OA were age- and sex-matched with patients with RA. Synovial fluids (SF) were centrifuged at 6,000 ×*g* for 15 min after they were aspirated from affected joints. Supernatants were stored at –80 °C. Synovitis severity measures, WBC counts in SF (cells/μl), and CRP level were obtained concurrently with SF sampling.

Assessment of synovitis severity via US

To determine synovitis levels, US of the joints was performed at the time of SF sampling. Sonographic evaluations of joints including GSUS and PDUS were performed as previously described.[2] Briefly, the degree of synovial hypertrophy was determined based on the GSUS score as follows: grade 0 = absence of synovial hypertrophy, grade 1 = minimal synovial hypertrophy, grade 2 = moderate synovial hypertrophy, and grade 3 = severe synovial hypertrophy. Synovial vascularity was evaluated based on a PDUS score as follows: grade 0 = absence of Doppler signal, grade 1 = minimal Doppler signal, grade 2 = moderate Doppler signal (greater than grade 1 but < 50% of Doppler signals in the total background), grade 3 = high Doppler signal (> 50% of Doppler signals in the total background). Active synovitis was determined as follows: no synovitis = GSUS grade < 2 and PDUS grade = 0; active synovitis = GSUS ≥ 2 and PDUS ≥ 1.

Determination of cell viability

RA-FLS viability was determined using MTT assays. In brief, RA-FLSs were seeded into 24-well plates at a density of 2×10^4 cells per well in DMEM supplemented with 10% FBS. After transfection with *MYH9* or control siRNA for 24 or 48 h, the cells were treated with MTT solution (Sigma–Aldrich) and then incubated for 3 h at 37 °C. The MTT solution was carefully removed and DMSO was added to solubilize formazan crystals. Optical density was then measured at a wavelength of 540 nm using a microplate reader (Molecular Devices, San Jose, CA, USA).

Immunocytochemistry

To visualize the lamellipodium-containing RA-FLSs, immunofluorescence staining of F-actin was performed on chamber slides. RA-FLSs were transfected with *MYH9* or control siRNA for 48 h or not transfected for non-muscle myosin II-inhibited conditions. RA-FLSs (5×10^3 cells per well) were seeded onto eight-well chamber slides (Nalgene Nunc International, Rochester, NY, USA) and incubated at 37 °C for 24 h in DMEM supplemented with 1% FBS containing TGFβ (10 ng/mL; Peprotech) or IL-1β (1 ng/mL; Thermo Fisher Scientific) or in DMEM supplemented with 5% FBS containing blebbistatin (Selleckchem, Houston, TX, USA) at 0.5 μM, 1 μM, or 5 μM, and DMSO as a vehicle control. For all experiments, cells were fixed with 4% paraformaldehyde in PBS (catalog 16320145; Wako Pure Chemicals, Osaka, Japan) for 20 min and permeabilized with 0.1% Triton X-100 in PBS for 5 min at room temperature. After washing with PBS twice, the cells were blocked with 1% BSA in PBS at room temperature for 60 min and incubated with Alexa Fluor 488-conjugated phalloidin (1:400; catalog A12379, Invitrogen, Waltham, MA, USA) alone or in combination with anti-MYH9 Ab (1:100; catalog 11128-1-AP; Proteintech, Rosemont, IL, USA) diluted in PBS at room temperature for 1 h. After washing with PBS, Alexa Flour 594-conjugated anti-rabbit IgG Ab (1:1000; catalog A21207; Life Technologies, Carlsbad, CA, USA) diluted in PBS was added to anti-MYH9 Ab-treated wells and incubated for 1 h at room temperature. After washing with PBS, nuclei were stained with DAPI (2 μg/mL; Roche). Coverslips were mounted on glass slides with Antifade Mounting Medium (Vector Laboratories, Newark, CA, USA). Cells were examined using a confocal microscope LSM 900 (Carl Zeiss, Oberkochen, Germany). Lamellipodium-containing cells were manually counted in three random fields.

Lee S, *et al. Ann Rheum Dis* 2023; 82:1035–1048. doi: 10.1136/ard-2022-223625

ELISA

CCL2, IL6, IL8, TGFβ, IL1β, and TNFα concentrations in RA (n = 80) and OA (n = 40) SFs were measured using ELISA kits (R&D systems). MYH9 levels in RA and OA SFs or culture supernatants of human monocytes and RA-FLSs were also determined using ELISA kits (MyBiosource, San Diego, CA, USA) according to the manufacturer's instructions.

Quantitative real-time PCR

Total RNA was isolated from RA-FLSs (8×10^5 cells per well in a 12-well plate) and human $CD14^+$ cells (1×10^6 cells per well in a 96-well flat-bottom plate) using RNeasy Mini kit (Qiagen, Hilden, Germany) according to the manufacturer's instructions. RNA was reverse transcribed into cDNA using RevertAid reverse transcriptase (Thermo Fisher Scientific). Real-time PCR was performed in a CFX96 real-time PCR system using SYBR Green PCR mixture (BioRad, Hercules, CA, USA). Data were normalized to that of *GAPDH* expression in each sample. Relative fold changes were calculated using the $2^{-\Delta\Delta Ct}$ algorithm [3]. The following primers were used for PCR amplification of target genes (forward and reverse): *MYH9*, 5′-CACTACCAACCTCATGGAAGAGG-3′ and 5′-TCCAACTCCTGCCTCTGCTTCT-3′; *GAPDH*, 5′-AAGGTGAAGGTCGGAGTCAA-3′ and 5′-AATGAAGGGGTCATTGATGG-3′.

Western blot analysis

RA-FLSs (1.0×10^5 cells per well in a six-well plate) and human $CD14^+$ cells (1.0×10^6 cells per well in a 96-well flat-bottom plate) were lysed in RIPA buffer (150 mM NaCl, 1% NP40, 0.5% sodium deoxycholate, 0.1% SDS, 50 mM Tris-HCl). Supernatants were then obtained after centrifugation at 12,000 ×*g* for 20 min at 4 °C. Protein concentrations were determined using a BCA assay kit (Bio-Rad). Proteins were resolved via SDS-PAGE on approximately 4–15% gradient acrylamide gels and transferred to PVDF membranes (0.45 μm pore size; GE healthcare Life science, Marlborough, MA, USA). Membranes were incubated with anti-MYH9 Ab (1:1000, catalog 3403S; Cell signaling technology), anti-myosin light chain (MLC) 2 Ab (1:1000; catalog 3672S; Cell signaling technology), anti-p-MLC Ab (Thr18/Ser19) (1:1000; clone E2J8F, catalog 95777; Cell signaling technology), anti-β-actin Ab (1:1000; clone C4, catalog sc-47778; Santa Cruz Biotechnology), anti-GAPDH Ab (1:1000; clone 6C5, catalog sc-32233; Santa Cruz Biotechnology), anti-Paxillin Ab (1:40,000; clone 349, catalog 610051; BD Biosciences, Franklin Lakes, NJ, USA), or anti-p-Paxillin (Tyr118) Ab (1:1000; clone E9U9F, catalog 69363; Cell signaling technology). Protein bands were visualized using an ECL kit (Santa Cruz Biotechnology).

Global proteomic profiling of the RA-FLS secretome

Protein extraction

RA-FLSs (2×10^6 cells per plate) were seeded into 150 mm^2 plates and cultured in DMEM supplemented with 10% FBS until 80% confluency was attained. Culture media were replaced with DMEM containing 1% insulin-transferrin-selenium with or without IL-1β (10 ng/mL; Thermo Fisher Scientific) or TNFα (10 ng/mL; Peprotech) and incubated at room temperature for 24 h. Culture supernatants from RA-FLSs were then centrifuged at 800 ×*g* for 5 min, pooled, and dried using a lyophilizer. Dried samples were homogenized in Tris buffer solution (25 mM Tris-HCl [pH 7.6], 150 mM NaCl, 1% NP-40, 1% sodium deoxycholate, 0.1% SDS; Thermo Fisher Scientific) with a protease inhibitor cocktail (1 mM AEBSF, 0.8 μM aprotinin, 50 μM bestatin, 15 μM E-64, 20 μM leupeptin, and 10 μM pepstatin A; Thermo Fisher Scientific; Calbiochem; Merck Millipore) and phosphatase inhibitor (10 mM sodium fluoride, 1 mM sodium orthovanadate, 2 mM β-glycerophosphate disodium salt hydrate, 2 mM sodium pyrophosphate decahydrate; Sigma–Aldrich). Homogenates were centrifuged at 13,000 rpm for 10 min at 4 °C. Supernatants were collected, and protein concentrations were determined using a bicinchoninic acid assay (23225; Thermo Fisher Scientific). Proteins were precipitated using 10% trichloroacetic acid at 4 °C overnight followed by centrifugation at 14,000 rpm for 10 min at 4 °C. The pellet was washed twice with cold acetone. The protein pellet was solubilized in 100 mM triethylammonium bicarbonate (TEAB).

Protein digestion

Proteins were digested using a filter-aided sample preparation method[4] with slight modifications. Briefly, proteins were reduced at 37 °C using SDT buffer (4% SDS in 0.1M Tris-HCl [pH 7.6] and 0.1 M DTT) for 45 min and then boiled at 95 °C for 10 min. Subsequently, protein samples were sonicated in a bath sonicator (vibra cell; Sonics) for 10 min and centrifuged at 16,000 ×*g* for 5 min. Protein samples were transferred to a membrane filter device (YM-30; Millipore) and mixed with 200 µl of 8 M urea in 0.1M Tris-HCl (pH 8.5). The device was centrifuged at 14,000 ×*g* for 60 min at 20 °C to remove SDS, which was repeated three times. Subsequently, proteins were alkylated with 100 µl of 50 mM iodoacetamide in 8 M urea for 25 min at room temperature in the dark, followed by centrifugation at 14,000 ×*g* for 30 min. The filter was washed with 200 µl of 8 M urea four times and then washed with 100 µl of 50 mM NH_4HCO_3 twice for buffer exchange. Trypsin (Promega) was added to proteins at an enzyme-to-protein ratio of 1: 50 (w/w). The filter device was placed in a thermomixer (Eppendorf) and incubated at 37 °C overnight. After the first digestion, the second digestion was performed using additional trypsin (1:100 enzyme-to-protein ratio) at 37 °C for 6 h. After digestion, tryptic peptides were eluted via centrifugation at 14,000 ×*g* for 30 min at 20 °C. After collecting the tryptic peptides, the filter was rinsed with 60 µl of 50 mM NH_4HCO_3 and centrifuged at 14,000 ×*g* for 20 min at 20 °C. The eluent was combined with the first eluent. The combined eluent was dried via vacuum centrifugation. Peptide concentration was determined using a BCA assay. The peptide sample was aliquoted into Eppendorf tubes (10 µg per tube), dried using vacuum centrifugation, and stored at –80 °C.

Peptide fractionation

To increase proteome coverage, mid-pH reverse phase liquid chromatography was performed at a flow rate of 0.5 ml/min using a 130-min gradient defined by solvent A (10 mM TEAB in water, pH 7.4) and solvent B (10 mM TEAB in 90% acetonitrile [pH 7.4]). The gradient used was as follows: 0% solvent B for 10 min, 0–5% solvent B in 5 min, 5–40% solvent B in 85 min, 40–70% solvent B in 5 min, 70% solvent B for 10 min, 70% to 5% solvent B in 10 min, and 0% solvent B for 10 min. A total of 96 fractions were collected from 15 min to 110 min and non-contiguously concatenated into 24 fractions by pooling four consecutive fractions (for instance, #1, 25, 49, and 73; and #2, 26, 50, and 73) from each of early (#1–24), first mid (#25–48), second mid (#49–72), and late (#73–96) sections of fractions. These 24 fractions were dried in a vacuum centrifuge concentrator and stored at -80°C until LC-MS/MS experiments.

LC–MS/MS analysis

Peptides (1 µg) from each of 24 fractions were dissolved in solvent A (2% acetonitrile and 0.1% formic acid). Nano-LC-MS/MS analyses were performed using a Q Exactive Mass Spectrometer (Thermo Fisher Scientific) equipped with an EASY-Spray Ion Source and coupled to an EASY-nLC 1000 chromatograph (Thermo Fisher Scientific). Peptides were loaded onto an Acclaim PepMap 100 pre-column (75 µm × 2 cm, C18, 3-µm particles, 100 Å pore size) and separated on an ES800 Easy-Spray column (50 cm × 75 µm inner diameter, PepMap C18, 3-µm particles, 100 Å pore size). A 120-minute gradient was used at a flow rate of 300 nl/min: from 2–40% solvent B (98% acetonitrile and 0.1% formic acid) over 90 min, from 40 to 80% solvent B over 10 min, 80% solvent B for 10 min, and 2% solvent B for 10 min. The temperature of the column was maintained at 35 °C. The electrospray voltage was set at 1.9 kV. MS precursor scans were acquired using the following settings: m/z range = 450–2000 Th, automated gain control (AGC) target value = 1.0 × 106, resolution = 70,000, and maximum ion injection time = 100 ms. For each MS scan, MS/MS data were acquired up to the ten-most abundant ions in a data-dependent mode using higher energy collisional dissociation with the following settings: normalized collision energy = 25, resolution = 17,500, AGC target value = 1.0 × 105, and maximum injection time = 50 ms.

LC-MS/MS data analysis

For each MS/MS dataset, post-experiment monoisotopic mass refinement was applied to accurately assign precursor masses to MS/MS spectra.[5] Resulting MS/MS spectra (i.e., mgf files) were subjected to a database search using the MS-GF+ search engine (v2017.01.13) [6] in

Lee S, *et al. Ann Rheum Dis* 2023; 82:1035–1048. doi: 10.1136/ard-2022-223625

a target decoy setting against the UniProt Human reference database (69,391 entries; February 2016) with the following parameters: precursor mass tolerance = 10 ppm; non-tryptic search; static modifications of carbamidomethylation (+ 57.021460 Da) to cysteine; variable modifications of oxidation (+ 15.994920 Da) to methionine; and carbamylation (+ 43.005810 Da) to N-termini. Search results from 24 MS/MS datasets were combined. Peptide spectrum matches were obtained using an FDR of 1%. The proteomics data were deposited to the ProteomeXchange with accession ID: PXD041077

Identification of subtypes of RA based on RNA-seq data

We first identified "expressed genes" as genes with TPM values of > 1 in more than 50% of the 152 patients with early and established RA using previously reported RNA-seq data (GSE89408).[7] To select genes with significant variations, we calculated median absolute deviations (MADs) and then selected the expressed genes with top 10% (MAD10), 20% (MAD20), and 30% (MAD30) of MADs. Next, we performed an orthogonal non-negative matrix factorization (ONMF) clustering [8] to the log2-fold-change matrix for the selected genes. ONMF was performed iteratively to produce consensus matrices which were used to calculate the cophenetic correlation coefficient for varying number of clusters (k = 2–6). Based on the cophenetic coefficient, we determined the final number of clusters (i.e., subtypes of RA) as previously described.[9]

PRM analysis of RA-FLS secretome in SFs

Depletion of abundant proteins

SF samples from 117 patients with RA and 45 patients with OA were centrifuged at 1,500 × *g* at room temperature for 15 min. Supernatants were filtered using 0.22 μm filters (Millipore, Burlington, MA, USA). Filtered samples were stored at –80 °C until further analysis. Samples were depleted to remove the 14 most abundant proteins (albumin, haptoglobin, transferrin, IgA, IgG, alpha 1-antitrypsin, alpha 2-antitrypsin, alpha 1-acid glycoprotein, apolipoprotein A1, apolipoprotein A2, complement C3, IgM, transthyretin, and fibrinogen) using the Human 14 multiple affinity removal spin cartridge (Agilent Technologies, Santa Clara, CA, USA). Depleted proteins were then washed and concentrated using 3 kDa MWCO filters (Amicon, Millipore). Depleted SF protein concentration was estimated using a bicinchoninic acid (BCA) assay and then subjected to filter-aided sample preparation digestion.

PRM analysis

PRM analyses were performed at the PRM mode using the same instruments used for global secretome profiling as described above. The inclusion list included *m*/*z* values, charge states, and retention time for 436 precursor ions of 151 target proteins, which were obtained from the global secretome analysis. The solvent compositions, gradient, and MS operation parameters for global secretome profiling were used here as described above. For quantification of target peptides, MS raw files were imported to Skyline (ver. 19.1.0). Precursor-product ion chromatograms were extracted, and peak areas were estimated using the targeted acquisition method.[10] Peptides with at least four fragment ions (dot-product score of ≥ 0.8) were used for peak area quantification. The abundance of a target peptide was then estimated as the sum of peak areas for individual fragment ions. Peak picking and integration boundaries were manually inspected. The PRM analysis data were deposited to the ProteomeXchange with accession ID: PXD041077

Identification of DEPs between RA and OA SFs

Peak areas of target peptides were converted to $\log_2$-areas and then normalized using the average chromatographic precursor intensity of each dataset. Using normalized peak areas while excluding zero values, differentially expressed peptides between different conditions were identified using a statistical method described previously.[11] For each peptide, two-tailed Student's t-test was applied to calculate the T-values for each comparison. To compute *p*-values for these T-values, we estimated an empirical distribution of T-values for the null hypothesis (a peptide is not differentially expressed) by performing 100,000 random permutations of the samples and then by applying the Gaussian kernel density estimation method to T-values obtained from random permutations.[12] For each peptide, FDRs were computed via the two-

Lee S, *et al. Ann Rheum Dis* 2023; 82:1035–1048. doi: 10.1136/ard-2022-223625

sided test using the empirical null distribution using Storey's method [13]. Differentially expressed peptides were selected based on an FDR ≤0.05 and absolute log_2-fold-changes of ≥ 0.33 (1.26-fold change), which corresponds to the mean of 2.5th and 97.5th percentiles of the null distribution of log_2-fold changes.

Subtype identification

We first defined "core samples" for each subtype as those with positive silhouette scores. To identify signature genes defining each subtype of the core samples, we compared log2-fold-changes in the core samples of the subtype with those of the other subtypes. The comparison was performed using a previously reported integrative statistical hypothesis testing method[14] that computes adjusted p values from two sample t-test and the median ratio test and then combines the adjusted p values into an overall p value. From the comparison, the final sets of gene signatures were selected as the genes with 1) overall p value of < 0.05, 2) median value of patients in the subtype larger than zero, and 3) median value of the patients in the subtype larger than that of the patients in the other subtypes. We next performed gene set enrichment analysis of the gene signatures for each subtype using ConsensusPathDB[15] and identified cellular pathways significantly (P < 0.01) represented by the gene signature.

Cell-type deconvolution analysis

A previously reported single cell RNA-seq dataset of synovial tissues from patients with RA was obtained from the Import database (accession ID: SDY998).[16] We used a unique molecular identifier (UMI) count matrix including molecules with ≥ 10 reads per UMI. The count matrix was filtered and analyzed using Seurat (version 4.1.0).[17] Quality control, normalization, and clustering were performed as described in the original study.[16] Briefly, we excluded low-quality cells (genes/cell <200, genes/cell > 1,810, cells/genes < 3, and cells with > 25% of molecules derived from mitochondrial genes). Gene expression levels were normalized across the filtered cells using default parameters of the Seurat-intrinsic LogNormalize function, and significantly variable genes were detected using the FindVariableFeatures function. The cells were then clustered using a default method in Seurat into five populations: fibroblasts, monocytes, T cells, B cells, and plasmablasts. Finally, for deconvolution of the five cell types in bulk mRNA data, CIBERSORTx was applied using the five cell populations and the count matrix with the absolute mode, S-mode batch correction, and 500 permutations.

Cell culture

RA-FLSs from passages 3 to 7 were isolated from synovial tissues of patients with RA as described previously.[18] RA-FLSs were incubated in DMEM (Welgene, Gyungsan, Korea) supplemented with 10% FBS (GIBCO). To induce *MYH9* gene expression and protein levels in RA-FLSs, cells were treated with 1% FBS DMEM containing IL-1β (10 ng/mL; Thermo Fisher Scientific, Waltham, MA, USA), TGFβ (10 ng/mL; Peprotech, Cranbury, NJ, USA), TNFα (10 ng/mL; R&D systems, Minneapolis, MN, USA), tunicamycin (10 and 50 μg/mL; Sigma–Aldrich, St. Louis, MO, USA), or thapsigargin (10 μM; Sigma–Aldrich). To acquire human monocytes, peripheral blood samples were obtained from healthy donors and mononuclear cells were isolated using Ficoll-Paque Plus (Cytiva, Marlborough, MA, USA). $CD14^+$ cells were isolated using anti-CD14 magnetic beads (Miltenyi Biotec, Bergisch Gladbach, Germany) according to the manufacturer's instructions and cultured in RPMI 1640 medium (Welgene) supplemented with 10% FBS. For stimulation, $CD14^+$ cells were treated with LPS (0.1 and 1 ng/mL; Sigma–Aldrich) TNFα (10 ng/mL; R&D systems) or IL-1β (1 ng/mL; Thermo Fisher Scientific, Waltham, MA, USA) in RPMI 1640 medium containing 10% FBS. All cells were incubated at 37 °C in an incubator supplied with 5% CO_2.

Knockdown of *MYH9* in RA-FLSs

RA-FLSs (1.0×10^5 cells per well) were seeded into 6-well culture plates and grown to 80% confluence within 24 h. These cells were transfected with 50 nM of control siRNA (Santa Cruz Biotechnology, Dallas, TX, USA) or *MYH9* siRNA (Dharmacon, Lafayette, CO, USA) for 24 or 48 h using Lipofectamine 3000 (Thermo Fisher Scientific) in an Opti-MEM medium (Thermo Fisher Scientific).

Lee S, *et al. Ann Rheum Dis* 2023; 82:1035–1048. doi: 10.1136/ard-2022-223625

Immunofluorescence staining of synovial tissues

RA synovial tissues embedded in OCT compound (Leica, Wetzlar, Germany) were cryo-sectioned (7 μm thickness). These tissue slides were fixed with cold methanol for 30 min at –20 °C and blocked with 10% normal donkey serum (Jackson ImmunoResearch) for 1 h at room temperature. Slides were then incubated with rabbit anti-MYH9 Ab (1:100; catalog 11128-1-AP, Proteintech), mouse anti-CD55 Ab (1:100; clone NaM16-4D3, catalog SC-51733, Santa Cruz Biotechnology), and mouse anti-CD68 Ab (1:100; clone KP1, catalog sc-20060; Santa Cruz Biotechnology) at 4 °C overnight. Each slide was washed three times with PBS and incubated with Alexa Flour 488-conjugated anti-rabbit IgG Ab (1:1000; catalog A21206, Life Technologies, Carlsbad, CA, USA) and Alexa Fluor 594-conjugated anti-mouse IgG Ab (1:1000; catalog A21203, Life technologies) at 4 °C for 2 h. Nuclei were stained with DAPI (2 μg/mL; Roche, Basel, Switzerland). After washing with PBS, coverslips were mounted on glass slides using Antifade Mounting Medium (Vector Laboratories). Images were obtained using a confocal microscope LSM 900 (Carl Zeiss).

Immunohistochemical analysis

Paraffin-embedded blocks of RA and OA synovial tissues were sectioned (5 μm thickness). These tissue sections were deparaffinized in xylene and rehydrated in a graded series of ethanol solutions. Antigen retrieval was performed by heating sections in citrate buffer using a microwave. Endogenous peroxidases were blocked by incubating sections in 3% H_2O_2 solution for 30 min at room temperature. These tissue sections were then blocked with 10% normal donkey serum (Jackson ImmunoResearch, West Grove, PA, USA) for 1 h at room temperature followed by incubation with rabbit anti-MYH9 Ab (1:3000; catalog 11128-1-AP, Proteintech) as a primary Ab at 4 °C overnight. Slides were washed three times with PBS and then incubated with a biotinylated anti-rabbit IgG secondary Ab (VECTASTAIN Elite ABC HRP kit; Vector Laboratories). A chromogenic substrate 3,3′-diaminobenzidine tetrahydrochloride (Vector Laboratories) was used for visualization. Nuclei were stained with Mayer hematoxylin. Slides were dehydrated, cleared, and mounted. Images were obtained using a Panoramic MIDI slide scanner (3DHISTECH, Budapest, Hungary).

Cell-spreading assays

For cell-spreading assays, RA-FLSs (1×10^4 cells per well) were dissociated by Accutase (catalog AT-104; Innovative Cell Technologies, San Diego, CA, USA) and seeded onto 12 mm-pi cover glasses (catalog 0111520; Marienfeld, Lauda-Königshofen, Germany) in a 24-well cell culture plate as previously described.[19]. The cover glasses were coated with fibronectin (20 μg/mL; catalog 1105147001; Merck, Kenilworth, NJ, USA) for 1 h at room temperature before used. The cells were incubated for 20, 40, 60 and 120 min in DMEM supplemented with 10% FBS. The spreading of the cells was stopped by fixation using 4% paraformaldehyde in PBS (Wako Pure Chemicals) for 20 min at room temperature. The staining process to visualize F-actin and MYH9 was the same as described above in **'Immunocytochemistry'**.

Cell migration and invasion assays

Wound migration of RA-FLSs was measured as described previously.[20] In brief, RA-FLSs were seeded onto 6-well plates at a density of 1.5×10^5 cells per well. After reaching approximately 90% confluence, the cells were transfected with *MYH9* or control siRNA (50 nM) for 24 h. After transfection, RA-FLSs were wounded by scratching cells with 200 μL sterile pipette tips and incubated with DMEM supplemented with 1% FBS containing IL-1β (1 ng/mL; Thermo Fisher Scientific) and TGFβ (10 ng/mL, Peprotech) for 12 h. For blebbistatin treatment conditions, RA-FLSs seeded onto 6-well plates were wounded as described above and treated with DMEM supplemented with 5% FBS containing 0.5 μM or 1 μM blebbistatin (Selleckchem) or with DMSO for 12 h. Cells were fixed with 4% paraformaldehyde (Wako Pure Chemicals) and stained with crystal violet solution. Images were captured using an EVOS Cell Imaging System (Thermo Fisher Scientific). Cells that migrated over the reference lines were manually counted in three random microscopic fields. Live cell images were obtained using a Lionheart FX automated microscope (Agilent). The percentage of closure area was calculated using the following formula:

$$Closure\ area\ \% = \left(\frac{A_{t=0} - A_{t=\Delta t}}{A_{t=0}}\right) \times 100\%$$

$A_{t=0}$ is the initial wound area and $A_{t=\Delta t}$ is the wound area after n hours of the initial scratch. For Matrigel invasion assays, RA-FLSs were transfected with *MYH9* or control siRNA (50 nM) for 24 h or were not transfected for non-muscle myosin II-inhibiting conditions. These cells were then detached and loaded (1.0×10^5 cells per well) into upper chambers of a 24-well Matrigel-coated Transwell plate (8 μm pore size; Corning, Corning, NY, USA) in a serum-free medium according to the manufacturer's instructions. Lower chambers of the Transwell plate were filled with DMEM supplemented with 1% FBS to attract cells in upper chambers for siRNA-treated conditions or were filled with DMEM supplemented with 5% FBS containing 0.5 μM or 1 μM blebbistatin (Selleckchem) or containing DMSO. Non-invading cells that remained at the top of the membrane surface were removed by scrubbing with a cotton swab after 12 h of incubation. Cells at the lower surface of the membrane were fixed with 4% paraformaldehyde in PBS (Wako Pure Chemicals), permeabilized with 100% methanol, and stained with 0.1% Crystal violet solution (Sigma–Aldrich). The number of migrating cells was manually counted in three random fields. Images were obtained using a Panoramic MIDI slide scanner (3DHISTECH).

Humanized synovitis in vivo model using SCID mice

Male SCID mice (age, 8 weeks) were obtained from Jackson laboratory and maintained in a specific pathogen-free facility. RA-FLSs were implanted together with normal human cartilages as described previously.[21] For developing a blebbistatin-injected model, RA-FLSs (2×10^6 cells per 100 μL) were resuspended in culture medium containing 1 μM of blebbistatin (Selleckchem) or DMSO and then implanted with human cartilage into the left flanks of SCID mice. For right flanks, only cartilages were implanted. SCID mice were then intraperitoneally injected with blebbistatin at a dose of 10 mg/kg twice a week for 60 d after the implantation. Implanted tissues were fixed with 4% paraformaldehyde in PBS (Wako Pure Chemicals) for 24 h and embedded in paraffin according to standard procedures. The degree of cartilage destruction was evaluated via hematoxylin and eosin (H&E) staining. Evaluation criteria were the same as those described in a previous study.[22] Briefly, scores were defined as follows: 0 = no invasions, 0.5 = invasion of one to two cell layers, 1 = invasion of three to five cell layers, 1.5 = invasion of three to five cell layers at three independent sites of the cartilage, 2 = invasion of 6 to 10 cell layers, 2.5 = invasion of 6 to 10 cell layers at three independent sites, 3 = invasion of >10 cell layers, 3.5 = invasion of >10 cell layers at two independent sites, 4 = invasion of >10 cell layers at three or more sites of the cartilage.

Induction and evaluation of methylated bovine serum albumin (mBSA)/IL-1β-induced arthritis in mice

Female C57BL/6 mice aged 6 weeks were obtained from Orientbio (Seongnam-si, Gyeonggi-do, Korea) and maintained in a specific pathogen-free facility. Methylated bovine serum albumin (mBSA) (200 μg; Sigma-Aldrich) was intra-articularly injected into the knee joints using a Hamilton syringe. From the day after the mBSA injection, recombinant IL-1β (250 ng; R&D systems) was administered subcutaneously into the hind footpads for three consecutive days. Then blebbistatin (10 mg/kg; Selleckchem) was intraperitoneally administered once a day for 10 days. Two weeks after mBSA injection, the mice were sacrificed, and the knee joint tissues were obtained. For histological analysis, the tissues were fixed in 4% paraformaldehyde (Biosesang, Seongnam-si, Gyeonggi-do, Korea) overnight at 4°C. The tissues were decalcified with Decalcifying Solution-Lite (Sigma-Aldrich) for 36 h at room temperature, sectioned, stained with hematoxylin and eosin (H&E), and then subjected to histological analysis. The degree of inflammation and bone destruction was evaluated for each joint as described previously: 0 = normal, 1 = weak, 2 = moderate, and 3 =severe. [20] The extent of cartilage destruction also was determined in Safranin O-stained tissues using a scale of 0 to 6 as described previously.[20]

Induction and evaluation of collagen-induced arthritis in mice

Male DBA/1 mice were immunized with bovine type II collagen (CII; catalog 20021, Chondrex, Woodinville, WA, USA) as described previously.[23] In brief, CII (4 mg/mL in 0.01 N acetic acid) was emulsified with complete Freund's adjuvant (catalog 7001, Chondrex) containing 4 mg/mL of

Lee S, et al. Ann Rheum Dis 2023; 82:1035–1048. doi: 10.1136/ard-2022-223625

heat-killed M. tuberculosis at a 1:1 ratio. The mice were intradermally injected with 50 μL of the emulsion (containing 100 μg of CII) at the base of the tail as a primary immunization. Two weeks after the primary immunization, the mice received booster injections with 100 μg of CII in incomplete Freund's adjuvant (catalog 7002, Chondrex) via footpad. From three weeks after primary immunization, the mice were intraperitoneally injected with blebbistaitn (Selleckchem) at a dose of 1 or 10 mg/kg twice a week for 3 weeks. The arthritis severity of each limb was assessed on a 0- to 4- point scale using visual inspection, as previously described.[18]

Statistical analyses

Data are presented as the mean ± SEM. To compare numerical data between groups, the Mann–Whitney U test and the Wilcoxon matched pairs tests were performed. Spearman correlation test was performed to analyze associations between cytokines and MYH9 levels in RA SFs. Two-way ANOVA followed by Sidak's post-test was used to evaluate significance between the curves of wound closure area at different time points. All statistical analyses were performed using GraphPad Prism software v8 (GraphPad Software, San Diego, CA, USA). The p-values < 0.05 were considered significant.

Lee S, *et al. Ann Rheum Dis* 2023; 82:1035–1048. doi: 10.1136/ard-2022-223625

Supplemental References

1. Aletaha D, Neogi T, Silman AJ, et al. 2010 rheumatoid arthritis classification criteria: an American College of Rheumatology/European League Against Rheumatism collaborative initiative. *Ann Rheum Dis* 2010;69:1580-8.

2. Kim JW, Kong JS, Lee S, et al. Angiogenic cytokines can reflect the synovitis severity and treatment response to biologics in rheumatoid arthritis. *Exp Mol Med* 2020;52:843-53.

3. Livak KJ, Schmittgen TD. Analysis of relative gene expression data using real-time quantitative PCR and the 2(-Delta Delta C(T)) Method. *Methods* 2001;25:402-8.

4. Wisniewski JR, Zougman A, Nagaraj N, et al. Universal sample preparation method for proteome analysis. *Nat Methods* 2009;6:359-62.

5. Shin B, Jung HJ, Hyung SW, et al. Postexperiment monoisotopic mass filtering and refinement (PE-MMR) of tandem mass spectrometric data increases accuracy of peptide identification in LC/MS/MS. *Mol Cell Proteomics* 2008;7:1124-34.

6. Kim S, Pevzner PA. MS-GF+ makes progress towards a universal database search tool for proteomics. *Nat Commun* 2014;5:5277.

7. Guo Y, Walsh AM, Fearon U, et al. CD40L-Dependent Pathway Is Active at Various Stages of Rheumatoid Arthritis Disease Progression. *J Immunol* 2017;198:4490-501.

8. Kim Y, Kim TK, Kim Y, et al. Principal network analysis: identification of subnetworks representing major dynamics using gene expression data. *Bioinformatics* 2011;27:391-8.

9. Mun DG, Bhin J, Kim S, et al. Proteogenomic Characterization of Human Early-Onset Gastric Cancer. *Cancer Cell* 2019;35:111-24 e10.

10. MacLean B, Tomazela DM, Shulman N, et al. Skyline: an open source document editor for creating and analyzing targeted proteomics experiments. *Bioinformatics* 2010;26:966-8.

11. Bolstad BM, Irizarry RA, Astrand M, et al. A comparison of normalization methods for high density oligonucleotide array data based on variance and bias. *Bioinformatics* 2003;19:185-93.

12. Bowman AW, Azzalini A. Applied smoothing techniques for data analysis : the kernel approach with S-Plus illustrations. Oxford: Oxford University Press 1997.

13. Storey JD, Tibshirani R. Statistical significance for genomewide studies. *Proc Natl Acad Sci U S A* 2003;100:9440-5.

14. Chae S, Ahn BY, Byun K, et al. A systems approach for decoding mitochondrial retrograde signaling pathways. *Sci Signal* 2013;6:rs4.

15. Herwig R, Hardt C, Lienhard M, et al. Analyzing and interpreting genome data at the network level with ConsensusPathDB. *Nat Protoc* 2016;11:1889-907.

16. Zhang F, Wei K, Slowikowski K, et al. Defining inflammatory cell states in rheumatoid arthritis joint synovial tissues by integrating single-cell transcriptomics and mass cytometry. *Nat Immunol* 2019;20:928-42.

17. Stuart T, Butler A, Hoffman P, et al. Comprehensive Integration of Single-Cell Data. *Cell* 2019;177:1888-902 e21.

18. Yoo SA, Park BH, Park GS, et al. Calcineurin is expressed and plays a critical role in inflammatory arthritis. *J Immunol* 2006;177:2681-90.

19. Beach JR, Hussey GS, Miller TE, et al. Myosin II isoform switching mediates invasiveness after TGF-beta-induced epithelial-mesenchymal transition. *Proc Natl Acad Sci U S A* 2011;108:17991-6.

20. Choi S, You S, Kim D, et al. Transcription factor NFAT5 promotes macrophage survival in rheumatoid arthritis. *The Journal of Clinical Investigation* 2017;127:954-69.

21. Pap T, Aupperle KR, Gay S, et al. Invasiveness of synovial fibroblasts is regulated by p53 in the SCID mouse in vivo model of cartilage invasion. *Arthritis Rheum* 2001;44:676-81.

22. Seemayer CA, Kuchen S, Kuenzler P, et al. Cartilage destruction mediated by synovial fibroblasts does not depend on proliferation in rheumatoid arthritis. *Am J Pathol* 2003;162:1549-57.

23. Brand DD, Latham KA, Rosloniec EF. Collagen-induced arthritis. *Nat Protoc* 2007;2:1269-75.

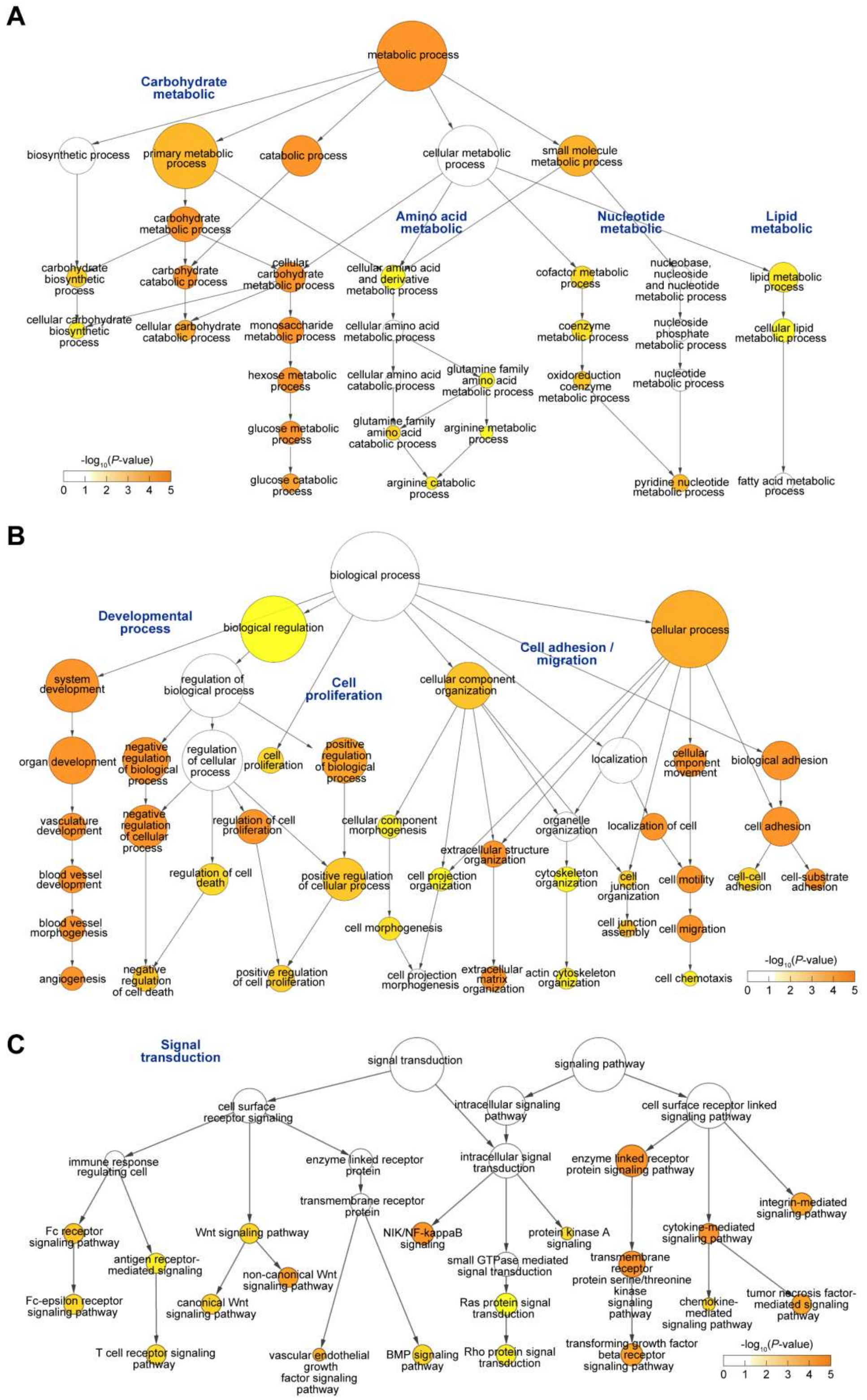

Lee S, *et al. Ann Rheum Dis* 2023; 82:1035–1048. doi: 10.1136/ard-2022-223625

Figure S1. BINGO GOBP networks illustrating hierarchical relationships among enriched metabolism-related GOBPs (**A**), pannus-related GOBPs (**B**), and signal transduction GOBPs (**C**) based on their GOBP levels. The arrow denotes a pair of higher and lower level GOBPs. Node size indicates the enrichment significance (*P*-value) as -log10(*P*-value). GOBP, Gene Ontology biological process.

Lee S, *et al. Ann Rheum Dis* 2023; 82:1035–1048. doi: 10.1136/ard-2022-223625

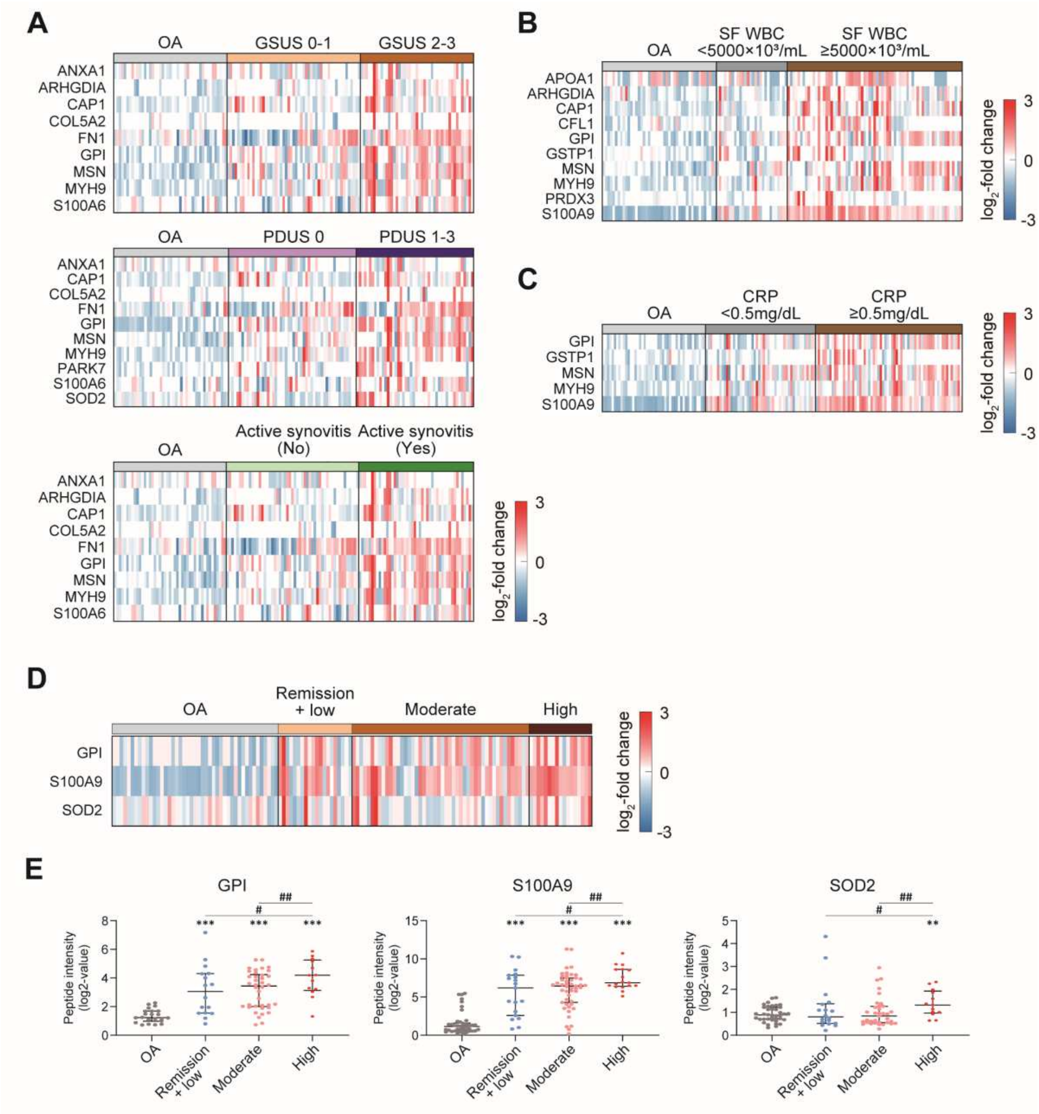

Figure S2. Differential expression of the secretory proteins in the synovial fluids between patients with OA and RA. (**A**) Heat maps of differentially expressed proteins in the comparisons of GSUS 0-1 versus GSUS 2–3, PDUS 0 versus PDUS 1–3, and active synovitis versus no active synovitis. (**B** and **C**) Heat maps from the comparisons of OA and the two RA groups separated on the basis of SF WBC count (B) and blood CRP level (C). (**D**) Heat maps of differentially expressed proteins in the comparison of OA, low RA activity (remission + low, $DAS28_{ESR} \leq 3.2$), moderate RA activity ($3.2 < DAS28_{ESR} \leq 5.1$), and high RA activity ($DAS28_{ESR} > 5.1$). (**E**) Graphs showing a differential expression of the three DEPs of GPI, S100A9, and SOD2 in osteoarthritis (OA) patients and the three groups of RA patients depending on RA activity based on $DAS28_{ESR}$. Top, middle, and bottom line on the graphs represent the 75th, 50th (median), and 25th percentile value of the abundance distributions of the indicated protein, respectively. ***p* < 0.01 and ****p* < 0.001 versus OA patients. #*p* < 0.05 and ##*p* < 0.01 between RA patients with low, moderate, and high RA activity. The *p* values were calculated as described in **Supplemental Methods**.

Lee S, *et al. Ann Rheum Dis* 2023; 82:1035–1048. doi: 10.1136/ard-2022-223625

CRP, C-reactive protein; GSUS, grey-scale ultrasonography; PDUS, power Doppler ultrasonography; SF, synovial fluid; OA, osteoarthritis; RA, rheumatoid arthritis; DAS, disease activity score; ESR, erythrocyte sedimentation rate; GPI, glucose-6-phosphate isomerase; S100A9, S100 calcium binding protein A9; and SOD2, superoxide dismutase.

Lee S, *et al. Ann Rheum Dis* 2023; 82:1035–1048. doi: 10.1136/ard-2022-223625

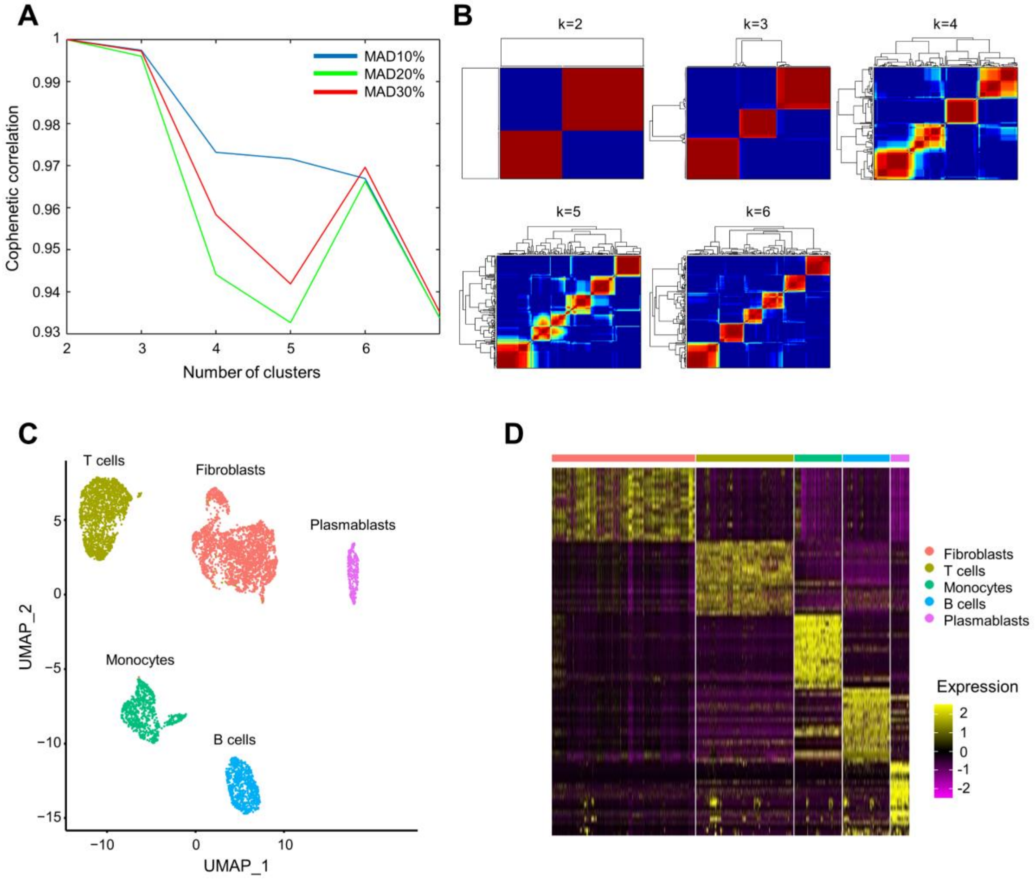

Figure S3. Analyses of bulk RNA-seq and single cell RNA-seq data. (**A**) A cophenetic correlation plot showing varying coefficients with a different number of clusters. Three plots are shown to summarize clustering results for 10, 20, and 30% of MADs. (**B**) Consensus clustering heat maps generated from 100 ONMF clustering trials using genes with MAD30%. Notably, cluster memberships for the three subtypes determined using genes with MAD30% were used for the following analyses. “Red” indicates that the corresponding pairs of samples were clustered mostly to the same cluster in the 100 clustering trails, whereas “blue” indicates that the pairs were rarely clustered together. (**C**) UMAP plot showing five clusters of cell types. (**D**) Heat map showing top 20 genes predominantly upregulated in each cell type. Top bar, the indicated cell types; color bar, gradient of log_2-fold-change of expression levels for each gene with respect to the media expression levels. MAD, median absolute deviations; UMAP, uniform manifold approximation and projection.

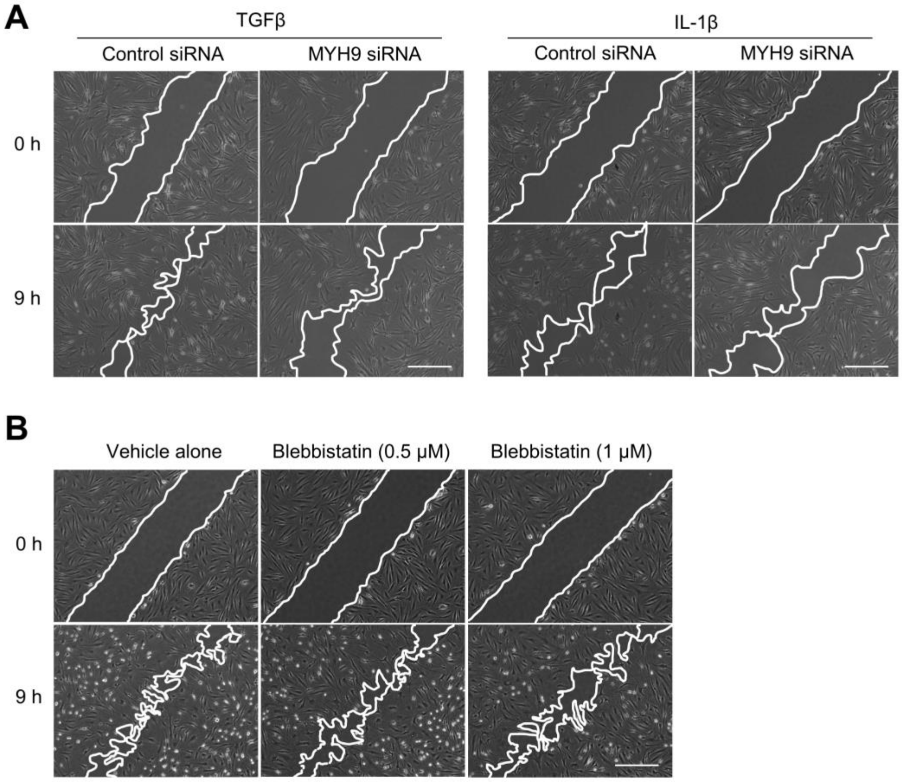

Figure S4. Real-time monitoring of the impairment of RA-FLS migration under MYH9-depleted and inhibited conditions. (**A**). RA-FLSs (n = 3) were transfected with *MYH9* siRNA (50 nM) for 24 h. The cells were then wounded using pipette tips. IL-1β (1 ng/mL) and TGFβ (10 ng/mL) in DMEM containing 1% FBS were added to wounded cells for 16 h. The wounded closure areas were monitored in real-time. Snapshot images at 9 h after incubation are presented. (**B**) Migration of wounded RA-FLSs (n = 3) in the presence of blebbistatin (0.5 and 1 μM) was evaluated as described in (A) and monitored in real-time for 16 h. Snapshot images at 9 h after incubation are presented. Live cell images and closure area analysis were obtained using a Lionheart FX microscope. Scale bars, 100 μm. MYH9, myosin heavy chain 9; RA-FLS, rheumatoid arthritis-fibroblast-like synoviocyte.

Lee S, *et al. Ann Rheum Dis* 2023; 82:1035–1048. doi: 10.1136/ard-2022-223625

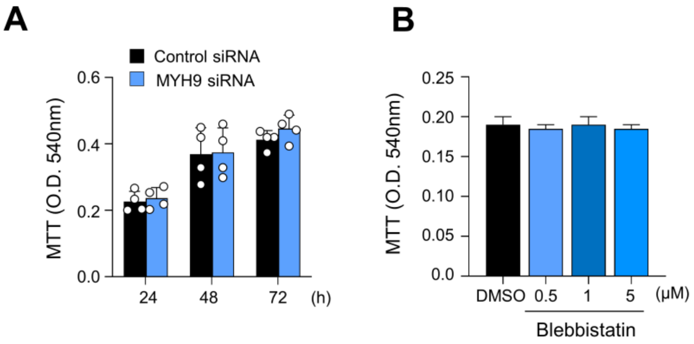

Figure S5. *MYH9* gene depletion and non-muscle myosin II inhibition using blebbistatin do not affect RA-FLS viability. RA-FLSs were transfected with *MYH9* siRNA (50 nM) for the indicated time (**A**, n = 4) or treated with blebbistatin (0.5, 1, and 5 μM) for 12 h (**B**, n = 2). Cell viability was determined using the MTT assay. MYH9, Myosin Heavy Chain 9; RA-FLS, rheumatoid arthritis-fibroblast-like synoviocyte.

Lee S, *et al. Ann Rheum Dis* 2023; 82:1035–1048. doi: 10.1136/ard-2022-223625

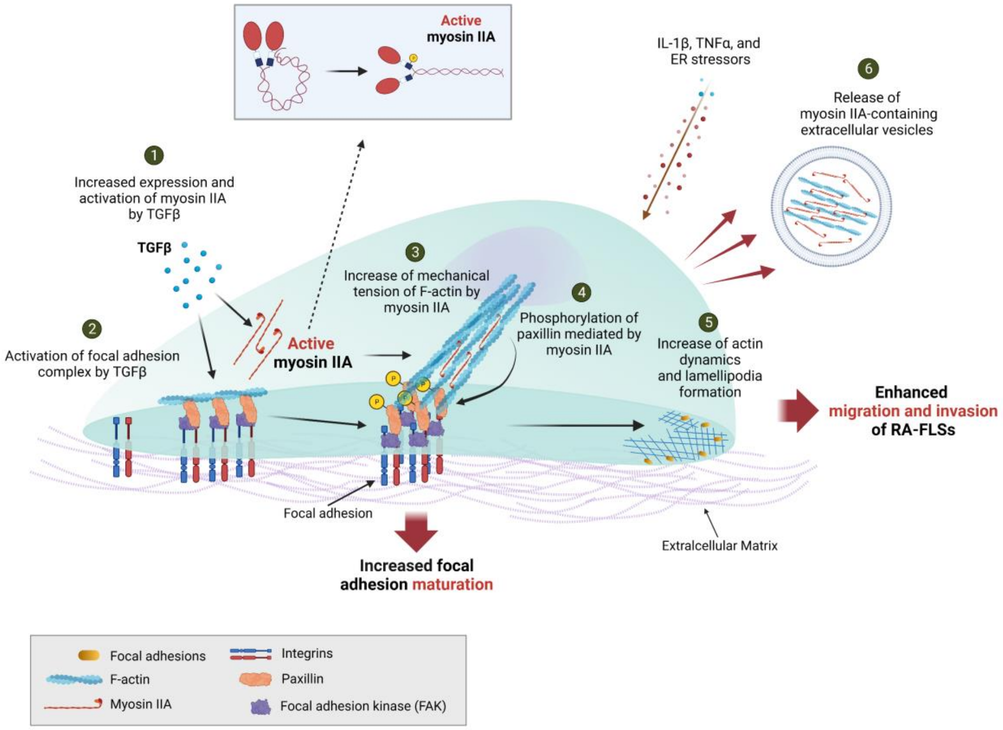

Figure S6. **Hypothetical model for the central role of MYH9 (heavy chain of myosin IIA) in adhesion, migration, and invasion of RA-FLSs.** 1) TGFβ increases expression of MYH9 and induces phosphorylation of myosin light chain to activate myosin IIA. 2) TGFβ may increase adhesion of RA-FLSs by directly activating FAK, a crucial mediator of focal adhesion. 3) Activated myosin IIA then binds to F-actin to promote intermolecular interaction between F-actins, increasing mechanical tension in the F-actin bundles and then enhancing maturation of focal adhesion complexes. 4) As a result, phosphorylation of paxillin, a key component of focal adhesion process, can be increased in manners dependent on MYH9 and FAK. 5) Increases in focal adhesion and actin dynamics mediated by myosin IIA result in the enhanced formation of lamellipodia, which can accelerate migration and invasion of RA-FLSs. 6) Additionally, microenvironments in the RA joints, including elevated levels of IL-1β, TNFα, and ER stress, appear to stimulate RA-FLSs to release myosin IIA, possibly in a form of extracellular vesicles, which could contribute to the spreading of RA-FLS aggressiveness to neighboring cells when delivered. RA-FLS, rheumatoid arthritis-fibroblast-like synoviocyte; FAK, focal adhesion kinase. Created with Biorender.com.

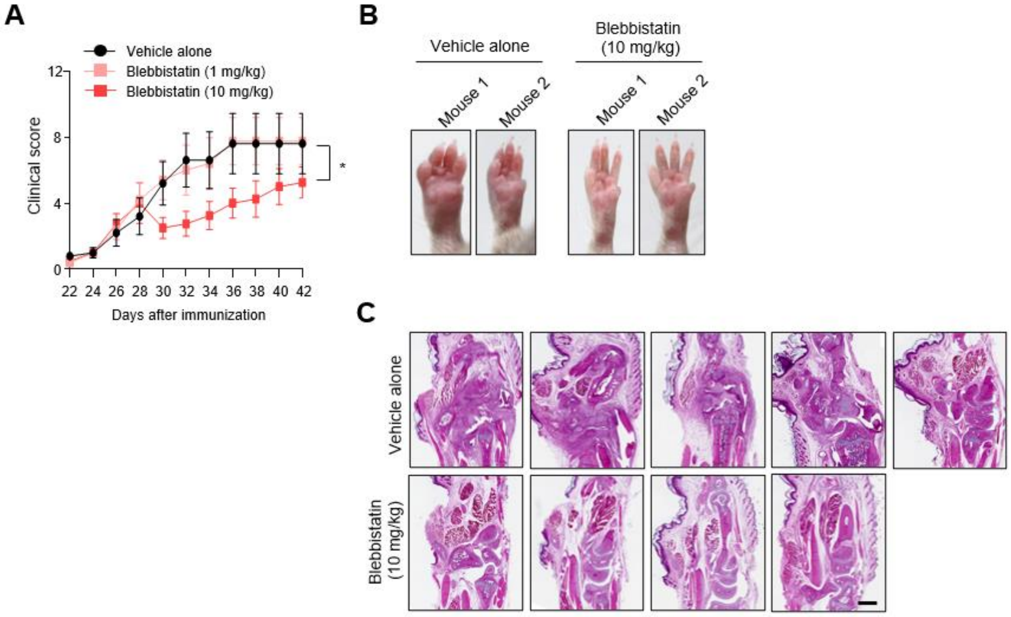

Figure S7. Therapeutic effects of blebbistatin on collagen-induced arthritis (CIA). (A) Bebbistatin suppression of clinical severity of CIA. From three weeks after primary immunization, mice were intraperitoneally injected with blebbistaitn twice a week for 3 weeks. Clinical severity was assessed by visual inspection in mice with CIA treated with vehicle alone (n = 5), blebbistatin at 1 mg/kg (n = 5), and blebbistatin at 10 mg/kg (n = 4). Data are presented as the mean ± SEM. The *p*-value was determined by two-way ANOVA followed by Sidak's post-test. $*p < 0.05$. (**B**) Representative photos showing the decrease in paw swelling and redness by blebbistatin. (**C**). H&E-staining of affected joints of mice treated with vehicle alone versus blebbistatin (10 mg/kg).

Lee S, *et al. Ann Rheum Dis* 2023; 82:1035–1048. doi: 10.1136/ard-2022-223625

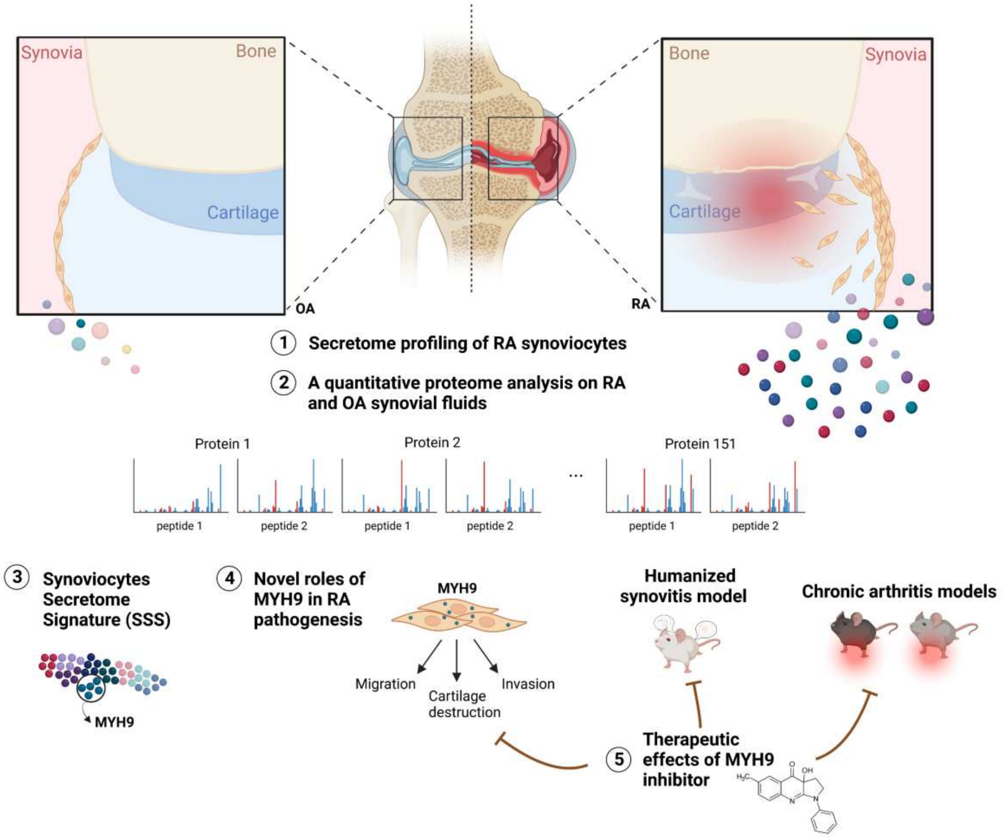

Figure S8. Schematic summary of this study demonstrating that MYH9, one of the "Synoviocyte Secretome Signature", plays a crucial role in migration and invasion of RA-FLSs, and it could be mitigated by blebbistatin, an inhibitor for MYH9. RA, rheumatoid arthritis; OA, osteoarthritis; MYH9, myosin heavy chain 9. Created with Biorender.com.

Lee S, *et al. Ann Rheum Dis* 2023; 82:1035–1048. doi: 10.1136/ard-2022-223625

Table S1. Selection of RA-FLS-derived secretory proteins related to "invasive pannus" among the whole RA-FLS secretome (n=843) using gene ontology biological process analysis

Pannus-related functions	Terms	Count
Cell migration & adhesion (179)	cell adhesion	118
	cell migration	49
	cytoskeleton organization	44
	chemotaxis	20
Extracellular matrix organization (177)	extracellular matrix organization	42
	carbohydrate catabolic process	30
	collagen fibril organization	15
	proteolysis	106
Cell proliferation & apoptosis (138)	regulation of cell proliferation	85
	anti-apoptosis	29
	regulation of apoptosis	73
Angiogenesis (46)	blood vessel development	46
	angiogenesis	29
Enriched signaling pathways (21)	TGFβ receptor signaling pathway	10
	Rho protein signal transduction	7
	Ras protein signal transduction	12
Total		409 (48.5% out of 843)

RA-FLS, rheumatoid arthritis-fibroblast-like synoviocytes.

Lee S, *et al. Ann Rheum Dis* 2023; 82:1035–1048. doi: 10.1136/ard-2022-223625

Table S2. Clinical information of patients with rheumatoid arthritis who provided SFs

**Variables**	**Total patients (n = 180)**
Age, mean ± SD, years	59.3 ± 13.5
Female sex, n (%)	149 (82.8)
Disease duration, mean ± SD, years	8.6 ± 8.3
RF, mean ± SD, IU/mL	208.9 ± 356.9
RF positive, n (%)	112 (62.2)
RF >3 ULN, n (%)	86 (47.8)
ACPA, mean ± SD, U/mL	161.8 ± 334.4
ACPA positive, n (%)	75 (41.7)
ACPA >3 ULN, n (%)	73 (40.6)
Blood WBC count, mean ± SD, /μL	7,964.8 ± 2,927.8
Blood neutrophil, mean ± SD, %	64.2 ± 11.1
Hemoglobin, mean ± SD, g/dL	12.4 ± 1.4
ESR, mean ± SD, mm/h	29.8 ± 27.8
CRP, mean ± SD, mg/dL	2.9 ± 6.4
WBC count in the SF, mean ± SD, /μL	15,783.1 ± 17,311.0
SF neutrophil, mean ± SD, %	54.5 ± 28.6
Glucocorticoid use, n (%)	134 (74.41)
Glucocorticoid dose, mean ± SD, mg*	4.5 ± 2.0
Methotrexate, n (%)	102 (56.7)
Leflunomide, n (%)	50 (27.8)
Sulfasalazine, n (%)	24 (13.3)
Hydroxychloroquine, n (%)	49 (27.2)
Tacrolimus, n (%)	27 (15.0)
biologic DMARDs, n (%)	48 (26.7)
Janus kinase inhibitor, n (%)	2 (1.1)
GSUS ≥2, n (%)	91 (50.6)
PDUS ≥1, n (%)	84 (46.7)
Active synovitis (GSUS ≥2 and PDUS ≥1), n (%)	61 (33.9)

*Prednisolone equivalent

RF, rheumatoid factor; ACPA, anti-citrullinated protein/peptide antibody; ULN, upper limit of normal; ESR, erythrocyte sedimentation rate; CRP, C-reactive protein; SF, synovial fluid; DMARDs, disease-modifying antirheumatic drugs; GSUS, grey-scale ultrasonography; PDUS, power doppler ultrasonography.

Table S3. Serum detectability of DEPs

Proteins	Synovitis	Synovial hypertrophy (GSUS)	Synovial vascularity (PDUS)	SF WBC	Blood CRP	References for blood detectability
PARK7			O			Blande M.F. et al., 2012
SOD2			O			Fernandez-Moreno M. et al., 2011
FN1	O	O	O			Yim S.Y. et al., 2015
COL5A2	O	O	O			The human protein atlas
ANXA1	O	O	O			Han G-H. et al., 2018
S100A6	O	O	O			Wei B-R. et al., 2009
MSN	O	O	O	O	O	The human protein atlas
GPI	O	O	O	O	O	Dai L. et al., 2010
MYH9	O	O	O	O	O	The human protein atlas
CAP1	O	O	O	O		Kakurina G.V. et al., 2017
RHGDIA	O	O		O		The human protein atlas
S100A9				O	O	Laouedj M. et al., 2017
GSTP1				O	O	Berendsen C.L. et al., 2000
APOA1				O		Ma C. et al., 2015
CFL1				O		Coskun A. et al., 2018
PRDX3				O		Ismail S. et al., 2015

Circles indicate that the DEPs show a significant difference in the comparisons between OA, mild RA, and severe RA groups for each clinical feature. References to serum detectability for the DEPs are listed.

CRP, C-reactive protein; SF, synovial fluid; GSUS, grey-scale ultrasonography; PDUS, power Doppler ultrasonography; DEP, differentially expressed protein.

Lee S, *et al. Ann Rheum Dis* 2023; 82:1035–1048. doi: 10.1136/ard-2022-223625

Movie S1A. **Real-time wound migration of RA-FLSs transfected with control siRNA under IL-1β-stimulated conditions**. RA-FLSs (n = 3) were transfected with control siRNA (10 nM) for 24 h. Then the cells were wounded with pipette tips and treated with IL-1β (1 ng/mL). Live cell images of the migrating cells were acquired for 16 h. Quantitative data and representative capture images of this movie are presented in figure 5E and supplemental material figure S5A, respectively.

Movie S1B. **Real-time wound migration of RA-FLSs transfected with control siRNA under TGFβ (10 ng/mL)-stimulated conditions**. RA-FLSs (n = 3) were transfected with control siRNA (10 nM) for 24 h. Quantitative data and representative capture images of this movie are presented in figure 5E and supplemental material figure S5A, respectively.

Movie S1C. Real-time wound migration of RA-FLSs transfected with *MYH9* siRNA under IL-1β-stimulated conditions. RA-FLSs (n = 3) were transfected with *MYH9* siRNA (10nM) for 24 h. Then the cells were wounded with pipette tips and treated with IL-1β (1 ng/mL). Quantitative data and representative capture images of this movie are presented in figure 5E and supplemental material figure S5A, respectively.

Movie S1D. Real-time wound migration of RA-FLSs (n = 3) transfected with *MYH9* siRNA under TGFβ (10 ng/mL)-stimulated conditions. Live cell images of the migrating cells were acquired for 16 h. Quantitative data and representative capture images of this movie are presented in figure 5E and supplemental material figure S5A, respectively.

Movie S1E. Real-time wound migration of RA-FLSs treated with vehicle control. RA-FLSs (n = 3) were wounded with pipette tips and added with medium containing vehicle control. Live cell images of the migrating cells were acquired for 16 h. Quantitative data and representative capture images of this movie are presented in figure 6D and supplemental material figure S5B, respectively.

Movie S1F. **Real-time wound migration of RA-FLSs (n = 3) treated with blebbistatin (0.5 μM).** Quantitative data and representative capture images of this movie are presented in figure 6D and supplemental material figure S5B, respectively.

Movie S1G. **Real-time wound migration of RA-FLSs (n = 3) treated with blebbistatin (1 μM).** Quantitative data and representative capture images of this movie are presented in figure 6D and supplemental material figure S5B, respectively.

Dataset S1. List of 843 RA-FLS secretome proteins. Data can be downloaded at the journal web page. RA-FLS, rheumatoid arthritis-fibroblast-like synoviocytes.

Lee S, *et al. Ann Rheum Dis* 2023; 82:1035–1048. doi: 10.1136/ard-2022-223625
